# Study on the mechanism of exogenous CaCl_2_ regulating cell growth and development to alleviate salt tolerance of alfalfa (*Medicago sativa*)

**DOI:** 10.3389/fpls.2025.1565723

**Published:** 2025-07-01

**Authors:** Huhu Liu, Ling Pan, Irshad Ahmad, Yuyu Bai, Sicong Shen, Renhuang Shi, Tingyang Xu, Ying Sun, Yang Gao, Bowen Kang, Yiwen Lu, Xiaoshan Wang

**Affiliations:** College of Animal Science and Technology, Yangzhou University, Yangzhou, China

**Keywords:** salt stress, calcium chloride, oxidative damage, morphological anatomy, transcriptome sequencing

## Abstract

Salt stress affects the growth and development of alfalfa. Calcium Chloride (CaCl_2_) plays a role in signal transduction and stabilizing cell membrane system in plant response to salt stress. However, the regulatory effect of CaCl_2_ on the growth and development of alfalfa cells under Sodium Chloride (NaCl) stress is not clear. This study analyzed the response and mitigation mechanism of adding 1mM CaCl_2_ to alfalfa seedlings under 120 mM NaCl stress. The results showed that the addition of CaCl_2_ increased the germination rate, vigor index and root length of alfalfa under salt stress. Secondly, the addition of CaCl_2_ reduced the activity of peroxidase, which led to the decrease of hydrogen peroxide (H2O2) and Malondialdehyde (MDA) content and catalase (CAT) activity. From the perspective of anatomical structure, the addition of CaCl_2_ under salt stress did not promote the elongation growth of alfalfa, which mainly reduced the area of root and leaf cells. Transcription sequencing showed that phenylpropanoid biosynthesis, Mitogen-Activated Protein Kinase (MAPK) signal transduction and photosynthesis pathways played a key role in alleviating NaCl stress when CaCl_2_ was added under salt stress. The up-regulation of genes including peroxidase (POD), chitinase (ChiB) and Light-Harvesting Complex (LHC) could regulate lignin accumulation and ethylene, abscisic acid and H2O2 transfer to defend against salt stress. In conclusion, CaCl_2_ can regulate the morphological physiology and antioxidant system of alfalfa, effectively alleviate the adverse effects of salt stress, and provide a basis for exploring the regulation of salt tolerance and the mitigation of salt stress

## Introduction

Salt stress is a key factor limiting plant growth and development (Shao et al., 2018). Soil salinization in abiotic stress is a global threat, and NaCl is the main cause of salt stress and limiting crop growth and development (Niu et al., 2022; Zhou et al., 2024). The area of saline soil in China is about 1.0 × 10 ha or 100 million hectares (Liu et al., 2021). Soil salinization will make it difficult for crop roots to absorb water, reduce the absorption efficiency of crops to nutrients, and also make soil tillage worse and reduce the beneficial microorganisms in the soil. The aeration and permeability of saline-alkali land are often poor and prone to stagnant water, thus delaying the surface soil temperature rise, reducing soil enzyme activity, changing soil physical and chemical properties to a certain extent, reducing soil fertility, and having a negative impact on crop growth and yield (Munns and Tester, 2008; Echeverria et al., 2021). Alfalfa is no exception as an important forage crop, by 2023, China ‘s alfalfa planting area will be about 1.33 million hectares (Solarte et al., 2021). Salt stress will interfere with the normal physiological and metabolic processes of alfalfa, resulting in many adverse effects such as limited growth and development, reduced yield and so on. The specific performance is as follows: high salt environment leads to osmotic stress and ion toxicity, hinders seed water absorption and embryo break through seed coat, resulting in the decrease of germination rate. Salt interferes with mitochondrial function and reduces capacity supply, resulting in seedling growth retardation, resulting in a decrease in this vigor index. However, Na^+^ accumulation destroys cell membrane integrity, inhibits cell division and elongation, and ultimately inhibits plant root growth (Xiong and Liu, 2018; Song et al., 2024).In order to reduce the harm caused by salt stress, plants will initiate a variety of defense mechanisms, including signal transduction, specific gene expression regulation and energy metabolism, and produce a variety of physiological reactions in plants, such as the establishment of antioxidant defense and induced enzyme scavenging system, including POD, CAT and other antioxidant enzymes, as well as H_2_O_2_ content to reduce oxidative damage to plants (Zhao et al., 2019; Li J. et al., 2023). At the same time, some exogenous substances have also been found to alleviate salt stress to a certain extent.

CaCl_2_ is one of the high-profile ones. Salt stress can destroy the integrity and stability of plant cell membranes, leading to the leakage of intracellular substances and affecting the normal function of cells. However, calcium ions in CaCl_2_ can bind to phospholipid molecules on cell membranes, enhance the structure of cell membranes, reduce the damage of salt ions to cell membranes, and maintain the fluidity and selective permeability of cell membranes (Guo et al., 2021). In the environment of salt stress, plants will absorb too much sodium ions, which will lead to the inhibition of the absorption of nutrient ions such as potassium ions and destroy the ion balance in cells. CaCl_2_ can reduce the absorption of sodium ions by plants through ion exchange, and promote the absorption and transport of beneficial ions such as potassium ions, so as to maintain the balance of intracellular ion concentration. Salt stress can induce a large amount of reactive oxygen species (ROS) in plants, such as superoxide anion, hydrogen peroxide, etc., which can cause oxidative damage to plant cells. CaCl_2_ can activate the antioxidant enzyme system in plants, such as superoxide dismutase (SOD), POD, CAT, etc., enhance the ability of plants to scavenge ROS and reduce oxidative damage (Wu et al., 2020; Köster et al., 2022). When plants respond to salt stress, the ability of exogenous application of CaCl_2_ to remove excess H_2_O_2_ in organs under salt stress is greatly enhanced. It can not only inhibit the absorption of Na^+^, but also act as a second messenger to activate related signal transduction pathways and gene expression, resulting in physiological changes in response to stress (Liu et al., 2022). When plants are subjected to high salinity, the increase of Ca^2+^ concentration can alleviate the inhibition of plant growth. In the field of plant stress resistance, Ca^2+^ can not only participate in a series of metabolic pathways, but also promote plant growth and development (Li et al., 2024). In conclusion, CaCl_2_ plays an important role in alleviating the effects of salt stress on plant morphology and anatomical structure, and its mechanism mainly includes maintaining cell membrane stability, regulating ion balance, participating in osmotic regulation, activating antioxidant enzyme system and regulating gene expression.

From the perspective of plants themselves, the morphological and anatomical structures of plants under salt stress will also change accordingly. These changes directly reflect the degree of stress suffered by plants and their own adaptation strategies. The in-depth study of plant adaptation to environmental stress through cell changes is helpful to further understand the growth and development of plants and the strategy of adapting to the environment (Jalili et al., 2022; Leal et al., 2022; Yue et al., 2022; Song et al., 2023).

With the development of molecular biology techniques, the analysis of the transcription level of alfalfa can explore the differential expression of many genes in the body when salt stress and exogenous addition of CaCl_2_ treatment, so as to clarify the molecular regulatory network in response to salt stress.

In view of the important position of alfalfa in agriculture and animal husbandry, in-depth study of its mechanism under salt stress, especially involving hydrogen peroxide, oxidative damage and CaCl_2_ mitigation, combined with morphological anatomy and transcriptome analysis, is of great theoretical and practical significance for revealing the salt tolerance mechanism of alfalfa, improving its growth adaptability in salinized soil and ensuring agricultural and animal husbandry production. The purpose of this study is to carry out in-depth discussion on these aspects, and lay a foundation for the cultivation and breeding and genetic improvement of alfalfa in the future.

## Materials and methods

### Experimental materials and growth environment

The experimental material was alfalfa ‘ Chifeng ‘ variety. (The test seeds were provided by the Pratacultural Laboratory of Yangzhou University, Jiangsu Province, China. The seeds were stored in a low temperature seed storage box at 4°C for 3 years.) The reagents used were CaCl_2_ and NaCl. Growth environment: temperature 25 ± 1°C, relative humidity 50%, day and night length of 16 hours and 8 hours of light incubator.

### NaCl and CaCl_2_ concentration screening and experimental treatment

The full and uniform alfalfa seeds were selected, disinfected with 75% anhydrous ethanol for 5 min, washed with distilled water for 3-5 times and dried. The NaCl concentration gradient was set as 0, 60, 120, 180 mM, and the CaCl_2_ concentration was set as 0, 0.5, 1.0, 1.5, 3.0 mM. Col, NaCl, CaCl_2_, NaCl + CaCl_2_ were used as the treatment groups. The filter paper and sponge were soaked with 25 ml treatment solution in the germination box, and 50 seeds were discharged with two layers of filter paper. Each treatment was repeated three times and cultured in a light incubator for 7 days.

### Measurement of morphological and physiological indexes

The number of germinated seeds (radicle length > 1 mm) was observed and recorded for 7 consecutive days. The relevant indicators were calculated as follows:


Germination rate (%)=number of normal germinated seeds/total number of test seeds×100%

Germination index (GI)=∑Gt/Dt,Gt is the number of seeds germinated on the same day,Dt is the corresponding germination days

Vigor index(VI )=SΣGt/Dt,S is the average root length

Root length of 10 seedlings were randomly measured. MDA content, CAT activity and POD activity were determined by spectrophotometry MDA content determination kit, CAT kit and POD kit of Suzhou Keming Co., Ltd. The H_2_O_2_ content of alfalfa was determined by H_2_O_2_ spectrophotometry kit. The remaining alfalfa was quickly frozen in liquid nitrogen and stored in a refrigerator at -80°C.

### Observation of anatomical structures

The roots and leaves of different treatment samples (3 replicates for each treatment) were placed in a 20-fold sample volume fixative for more than 24 h. The tip 1-3 mm was cut transversely and longitudinally, stained with toluidine blue, and observed under a microscope.

### RNA-seq analysis

Total RNA was obtained from alfalfa seedlings using the RNA Kit (Tiangen, Beijing, China) guidelines. Transcriptome sequencing used 3 biological replicates. Oligonucleotides (dT) were then added to RNA, and cDNA was synthesized by mixing fragment buffers. After that, PCR amplification products were used and end-to-end sequencing was performed using illumina NovaSeq 6000 (illumina, USA). Total RNA was extracted using the Tiangen Polysaccharide and Polyphenol Kit (QIAGEN, Germany). Quantitative analysis, gene difference analysis, Gene Ontology (GO) and Kyoto Encyclopedia of Genes and Genomes (KEGG) enrichment analysis were performed on the transcriptome data obtained, and then the clustering tree was constructed by Weighted Gene Co-Expression Network Analysis (WGCNA) analysis and the modules were divided according to the correlation of gene expression. For the species not contained in the database, we first applied blast to align the sequences in the target gene set to the protein sequences of the reference species contained in the Search Tool for the Retrieval of Interacting Genes/Proteins (STRING) database, and used the protein interaction relationship of the reference species on the alignment to construct the interaction network.

### RT-qPCR analysis

The quality and concentration of the extracted RNA were detected by 1% agarose gel electrophoresis and Agilent2100. 5 μl diluted to 100 ng/μl of the RNA solution to be tested and 1 μl of 6xLoading Buffer were drawn with a pipet gun, placed in a 200 μl centrifuge tube without RNA enzyme, fully mixed, and added to the prepared 1.5% agarose gel. Under the conditions of 120 V voltage and 100 mA current. Electrophoresis for 30-40 minutes, watching and taking pictures in the gel imaging system, and judging RNA quality according to the position and brightness of the band. The fluorescence quantitative primers were designed by primer premier 5.0, and Ms Actin (XM_024778429.2) was used as the internal reference. The differentially expressed genes with higher expression levels in the KEGG pathway with significant changes were selected for verification. The extracted RNA was reversed into cDNA (**Supplementary Tables S1**, **S2**) using the Tiangen reverse transcription kit, and verified on a fluorescence quantitative PCR instrument. The reaction system and reaction procedure (**Supplementary Tables S3**, **S4**). Each treatment had 3 biological replicates and 3 technical replicates, and the relative expression of the gene was calculated according to the 2^-ΔΔCT^ method.

### Statistical analysis

Statistical analysis SPSS 19.0 and Microsoft Excel 2018 were used for statistical analysis. The data were analyzed by one-way analysis of variance (ANOVA), and the least significant difference (LSD) test was used to compare the differences between treatments. Tukey multiple comparison test was used, and the difference was statistically significant (*P*< 0.05). GraphpadPism9.5 and R language program were used for drawing.

## Results

### Effects of exogenous CaCl_2_ on seed germination and physiological characteristics of alfalfa

**Figure 1:**
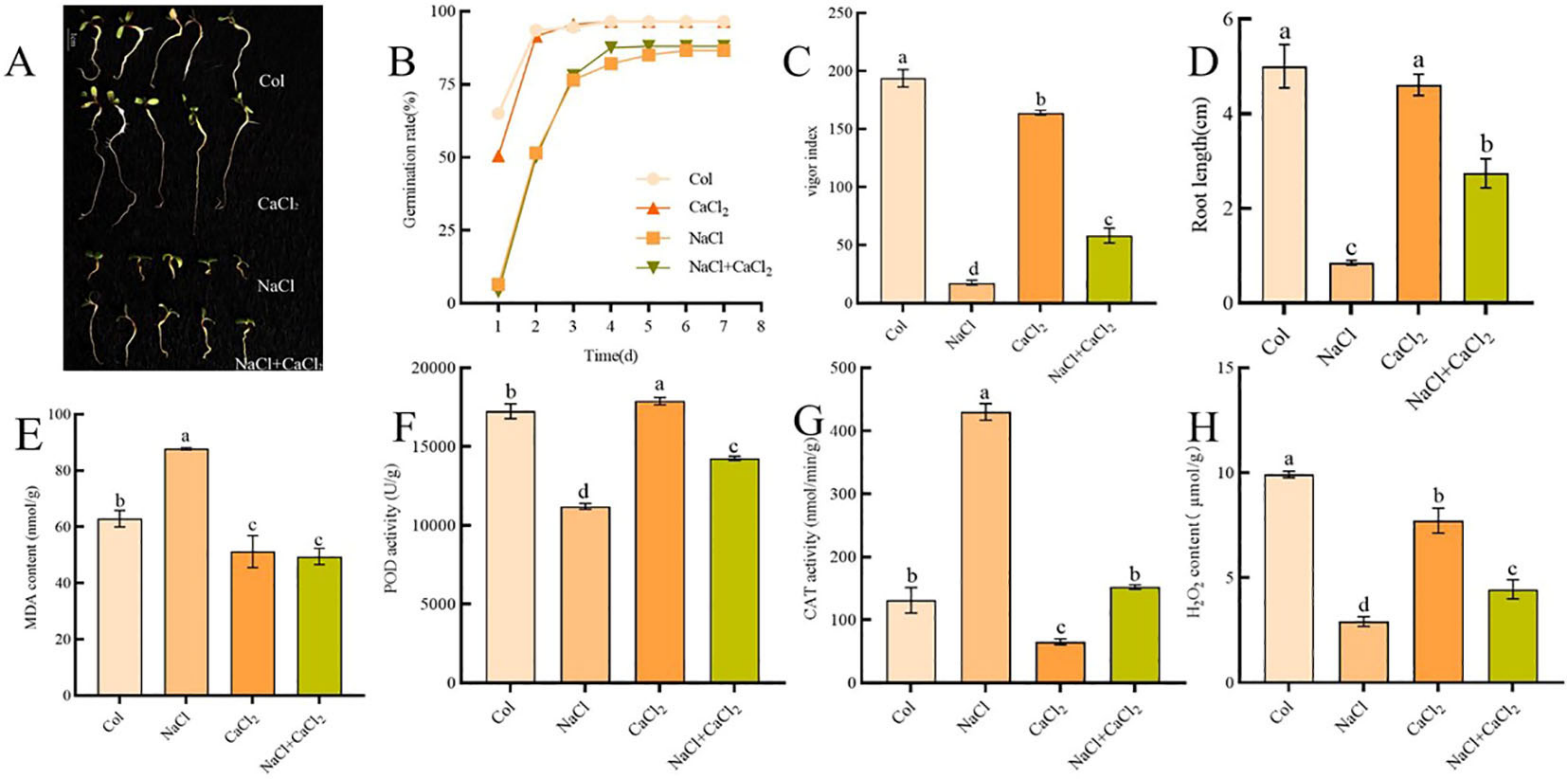
Analysis of germination morphology and physiological characteristics of alfalfa seeds treated with NaCl, CaCl_2_ and NaCl + CaCl_2_ for 7 days; **(A)** Morphological characteristics of alfalfa seeds after 7 days of germination; **(B)** seed germination rate; **(C)** seed vigor index; **(D)** Root length; the contents of MDA, POD, CAT and H_2_O_2_ were shown in **(E–H)**; (Different letters on the histogram represent the significant differences between the treatment group and the control group determined by the LSD test, and the letters a-d represent the significant range from p< 0.001 to p< 0.05 (the same below).

In order to test the salt tolerance of alfalfa seeds during germination, the germination test of alfalfa seeds was carried out under the conditions of NaCl concentration of 0,60,120,180 mM. It was found that the germination of alfalfa seeds was not significantly inhibited at 60 mM NaCl concentration, and the germination of alfalfa seeds was significantly inhibited at 120 mM NaCl concentration. When the NaCl concentration was 180 mM, the alfalfa seedlings were seriously damaged. Exogenous CaCl_2_ was added at 120 mM NaCl stress concentration, and the concentration of CaCl_2_ was 0.5,1.0,1.5,3.0 mM. After 7 days of seed germination, it was found that 1.0mM CaCl_2_ had a significant mitigation effect on salt stress of seedlings (**Supplementary Figures S1A, B**). The germination rate was 91.29%, 67.79%, 89.07% and 69.07% respectively on the 7th day of control, NaCl, CaCl_2_ and NaCl + CaCl_2_ treatment (**Figures 1A, B**). The seed vigor decreased significantly under NaCl treatment, and the seed vigor index increased significantly under CaCl_2_ treatment. The seed vigor indexes under NaCl, CaCl_2_ and NaCl + CaCl_2_ treatments were 8.45%, 83.63% and 26.86% of the control, respectively (**Figure 1C**). The root lengths under NaCl and NaCl + CaCl_2_ treatments were 17.13% and 54.84% of the control, respectively (**Figure 1D**). NaCl treatment increased MDA content and CAT activity, and decreased POD activity and H_2_O_2_ content. NaCl+CaCl_2_ alleviated the increase of MDA content, CAT activity and the decrease of POD activity and H_2_O_2_ content (**Figures 1E–H**).

### Effects of exogenous CaCl_2_ on morphological anatomy of alfalfa

**Figure 2:**
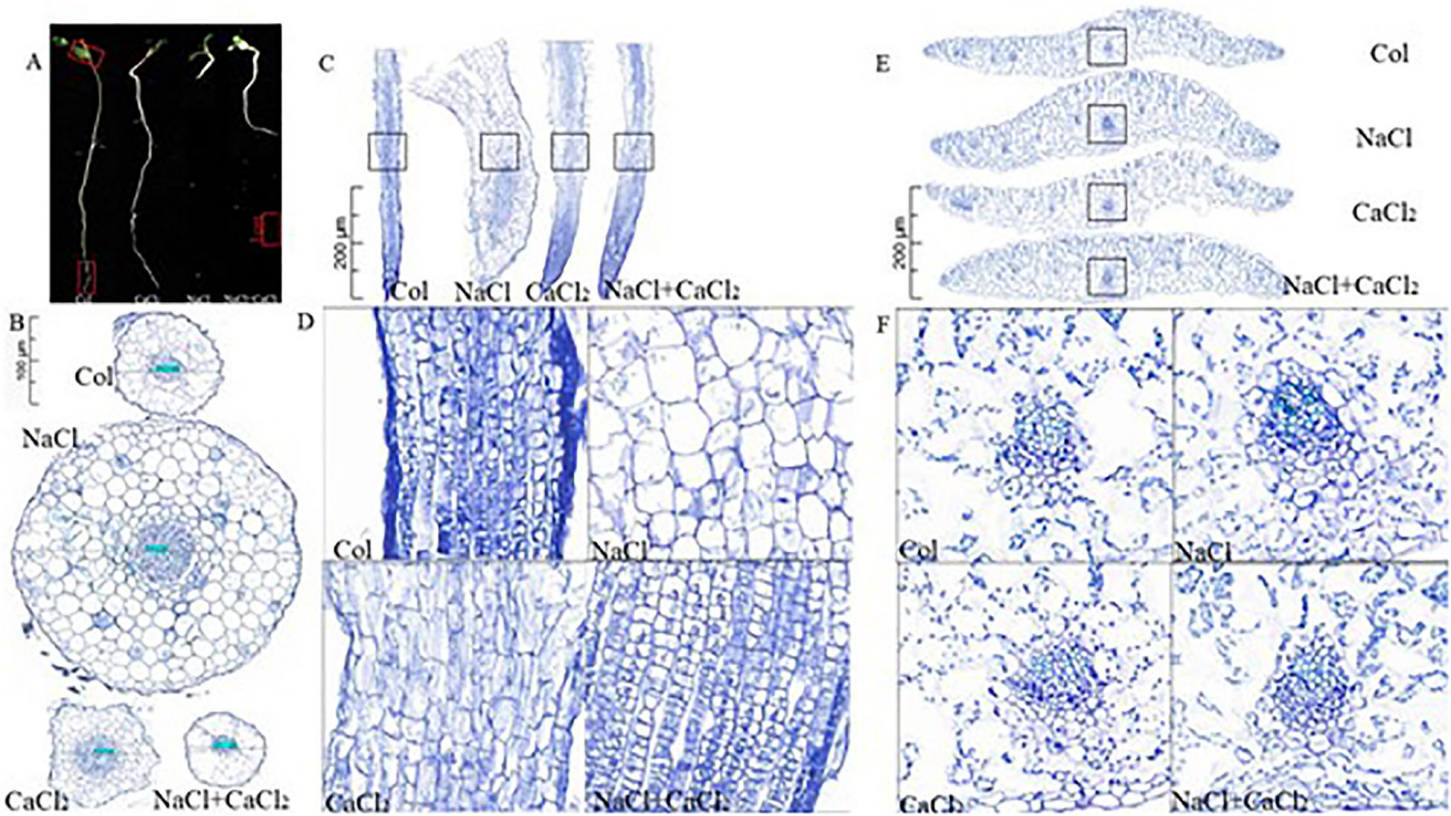
The morphological anatomy of alfalfa seeds under the same ratio of NaCl, CaCl_2_ and NaCl + CaCl_2_ treatment. **(A)** Morphological anatomy sampling phenotype diagram; **(B)** Anatomical cross-sectional diagram of root morphology; **(C)** Sampling diagram of root morphological anatomy longitudinal section, **(D)** Sampling diagram of root morphological anatomy longitudinal section, **(E)** Sampling diagram of leaf morphological anatomy transverse section, **(F)** Sampling diagram of root morphological anatomy cross section.

**Figure 3:**
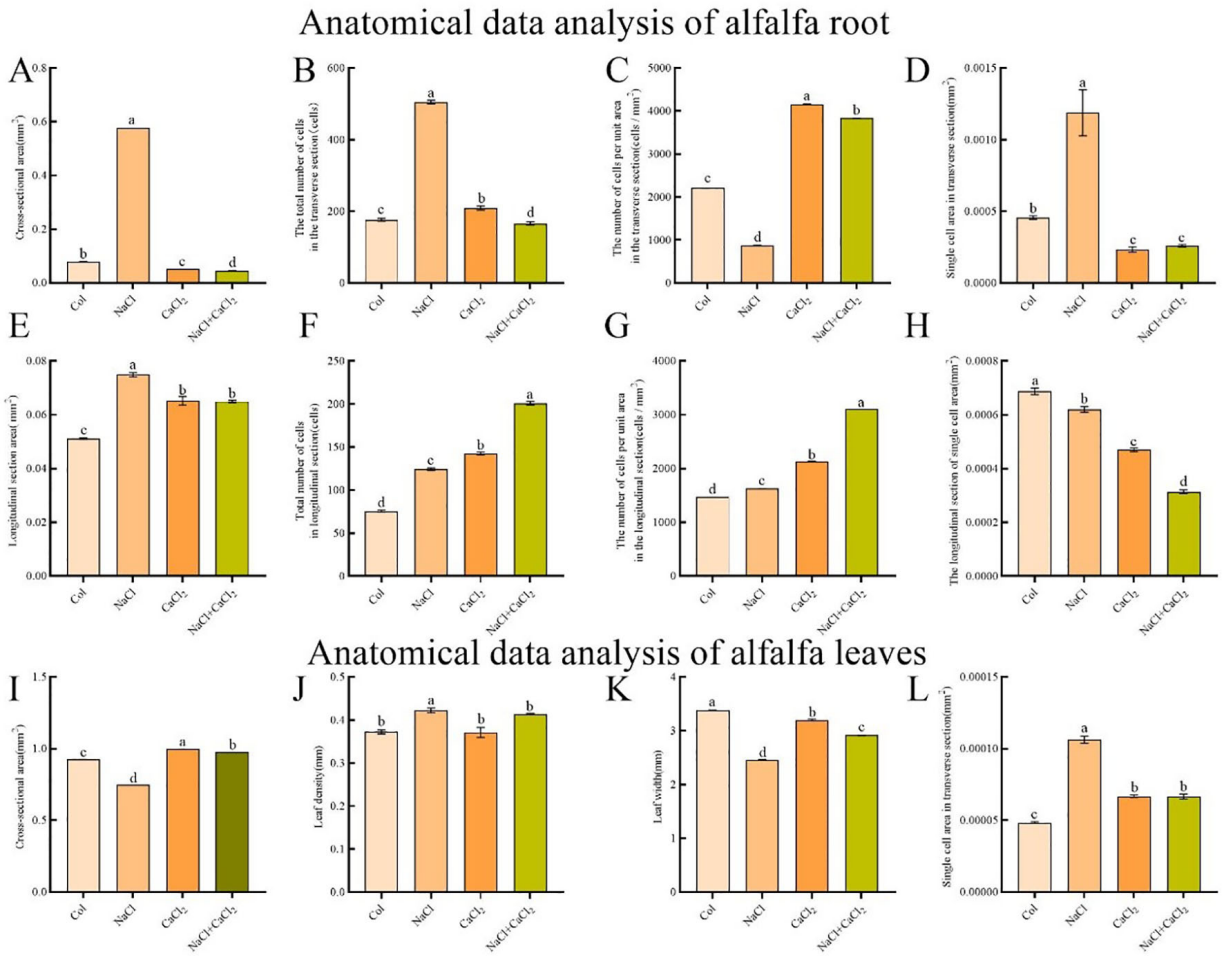
Analysis of root anatomical data of alfalfa seeds germinated for 7 days under NaCl, CaCl_2_ and NaCl + CaCl_2_ treatments. **(A–D)** were root anatomical cross-sectional area (mm^2^), total number of cells (cells), number of cells per unit area (cells/mm^2^) and single cell area (mm^2^). **(E–H)** were root anatomical longitudinal section area (mm^2^), total number of cells (cells), number of cells per unit area (cells/mm^2^) and single cell area (mm^2^). **(I–L)** were the total area of alfalfa leaf cross-section (mm^2^), leaf thickness (mm), leaf width (mm) and leaf single cell area (mm^2^).

Under 120 mM NaCl treatment, the root elongation of alfalfa seedlings was significantly inhibited, and the lateral growth of roots was promoted (**Figures 2A–C**). Compared with the control, the cross-sectional area and longitudinal section area of roots under NaCl treatment increased by 6.28 multiple and 0.46 multiple, respectively. Compared with NaCl treatment, the cross-sectional area and longitudinal section area of roots under NaCl + CaCl_2_ treatment decreased by 92.32% and 13.36% (**Figures 3A, E**). Compared with the control, the total number of cells in the cross section and the total number of cells in the longitudinal section of the root under NaCl treatment increased by 1.86 multiple and 0.64 multiple, respectively. Compared with NaCl treatment, the total number of cells in the cross section of the root under NaCl + CaCl_2_ treatment decreased by 67.06%, and the total number of cells in the longitudinal section increased by 0.62 multiple (**Figures 3B, F**). Compared with the control, the number of cells per unit area of root cross section under NaCl treatment decreased by 60.69%, and the number of cells per unit area of root cross section under NaCl + CaCl_2_ treatment increased by 3.39 multiple compared with NaCl treatment (**Figure 3C**). Compared with the control, the number of cells per unit area of root longitudinal section under NaCl treatment increased by 0.1 times, and the number of cells per unit area of root longitudinal section under NaCl + CaCl_2_ treatment increased by 0.91 times compared with NaCl treatment (**Figure 3G**). Compared with the control, the single cell area of root cross section under NaCl treatment increased by 1.6 times, and the single cell area of root longitudinal section decreased by 9.74%. Compared with NaCl treatment, the single cell area of root cross section and longitudinal section under NaCl + CaCl_2_ treatment decreased by 78.06% and 49.3%, respectively (**Figures 3D, H**). Anatomical observation of leaves showed that the leaves of alfalfa became thicker and narrower under NaCl treatment, the total area of leaf anatomical surface became smaller, and the leaf cells became larger, while the addition of CaCl_2_ alleviated the thickening and narrowing of leaves and the increase of cells caused by NaCl treatment (**Figures 2E, F**, **Figures 3I–L**).

### Comparison of differentially expressed DEGs in alfalfa seedlings under NaCl stress and NaCl stress with exogenous addition of CaCl_2_

**Figure 4:**
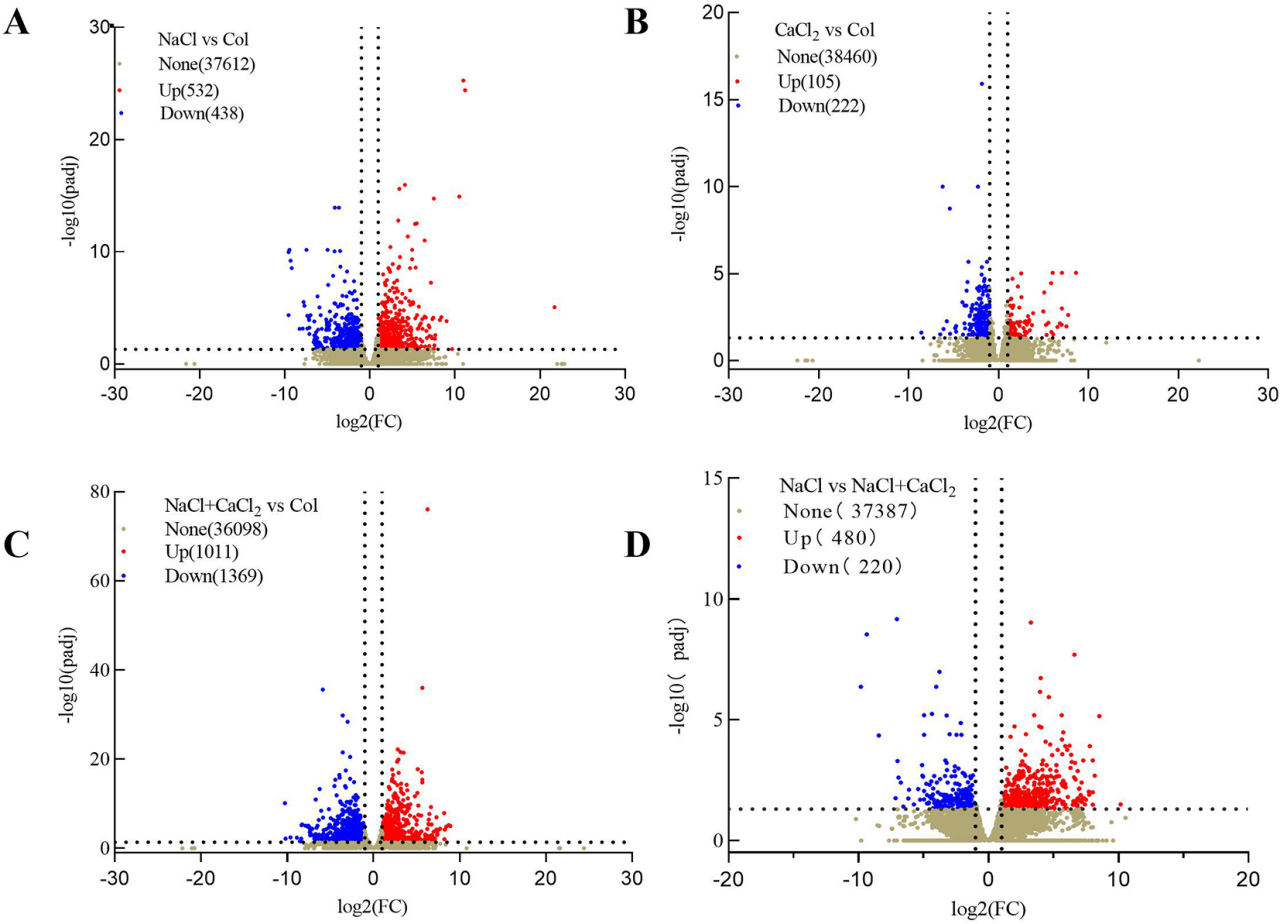
Differentially expressed DEGs volcano plots of alfalfa seeds germinated for 7 days under NaCl, CaCl_2_, NaCl + CaCl_2_ treatments **(A)** NaCl vs Col, **(B)** CaCl_2_ vs Col, **(C)** NaCl + CaCl_2_ vs Col and **(D)** NaCl vs NaCl + CaCl_2_. (Red indicates up-regulated DEGs, and green indicates down-regulated DEGs. The x-axis represents the log2-fold change conversion of the value, and the y-axis represents the significant value after the-log10 conversion. The dotted line represents the threshold or critical line of the differential DEGs screening criteria. None indicates no statistical significance, Up indicates up-regulated genes, Down indicates down-regulated genes).

In order to detect the molecular mechanism of morphological differences in alfalfa seedlings under NaCl stress and NaCl stress with exogenous CaCl_2_ addition, the illumina NovaSeq 6000 platform was used to perform transcriptional sequencing analysis on 7-day seedlings of each treatment, and the correlation and principal component analysis of the sequencing sample data were analyzed (**Supplementary Table S5**; **Supplementary Figures S3A, B**). The results show that the data quality is high and can be used for subsequent mechanism analysis. A total of 79.1 G pure data and 58968 DEGs were obtained by transcriptome sequencing of 12 samples. Each sample generated an average of 6.64 G data, obtained an average of 43.75~46.94 (million) Raw Reads, and obtained an average of 42.65~45.76 (million) after filtering (**Supplementary Table S6**). In the sequencing results of NaCl vs Col, a total of 970 differentially expressed DEGs were detected, of which 532 were up-regulated and 438 were down-regulated (**Figure 4A**). In the sequencing results of CaCl_2_ vs Col, a total of 327 differential DEGs were detected, of which 105 were up-regulated and 222 were down-regulated (**Figure 4B**). In the sequencing results of NaCl + CaCl_2_ vs Col, a total of 2380 differential DEGs were detected, of which 1011 were up-regulated and 1369 were down-regulated (**Figure 4C**). In the sequencing results of NaCl vs NaCl + CaCl_2_, a total of 700 differentially expressed DEGs were detected, of which 480 were up-regulated and 220 were down-regulated (**Figure 4D**). A total of 249 differential DEGs were screened out in the combination of NaCl vs NaCl + CaCl_2_ (**Supplementary Figure S3C**).

### GO and KEGG enrichment analysis of differentially expressed DEGs in alfalfa seedlings under NaCl stress and exogenous addition of CaCl_2_ under NaCl stress

**Figure 5:**
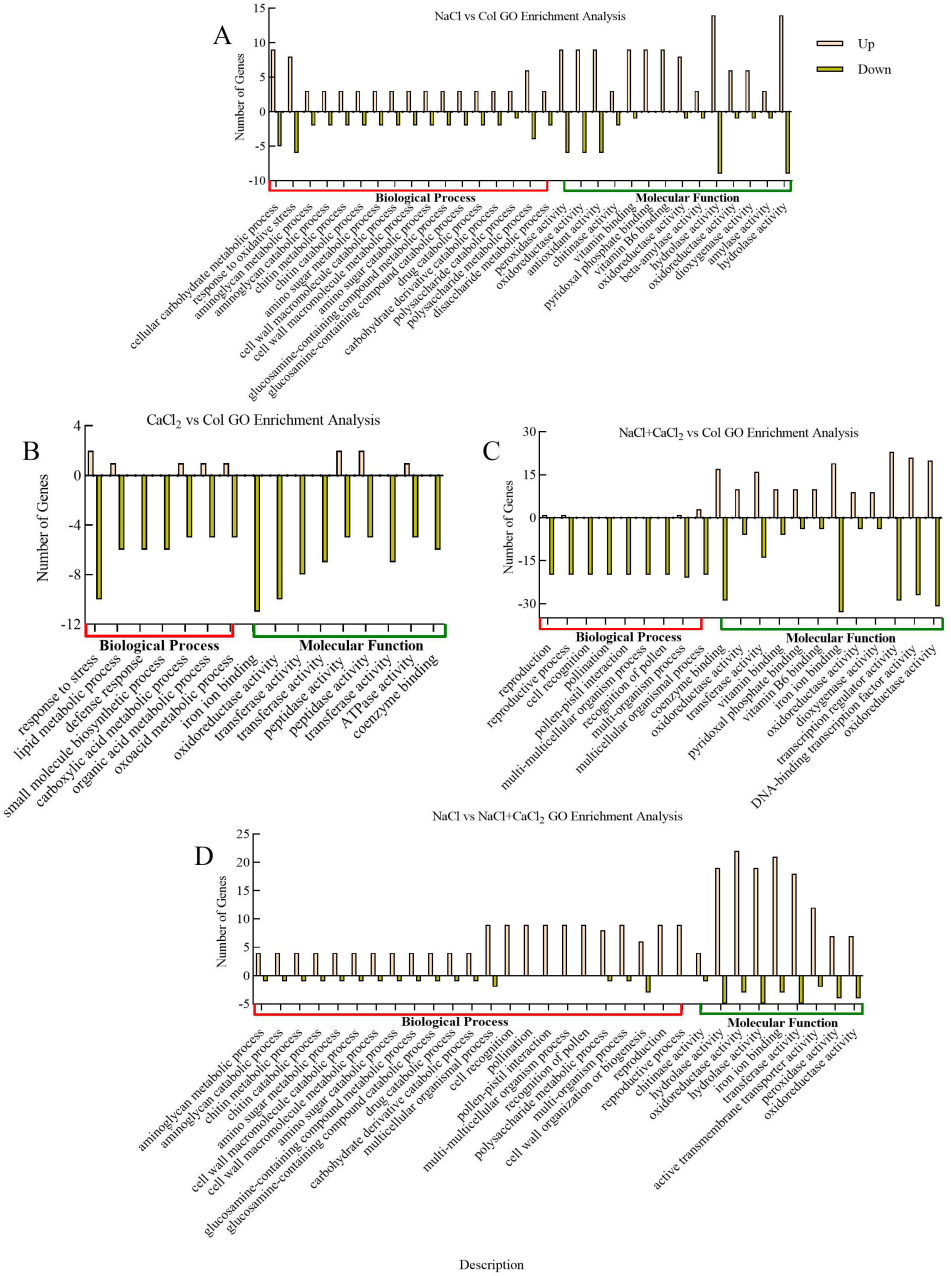
GO enrichment analysis of differentially expressed DEGs in **(A)** NaCl vs Col, **(B)** CaCl_2_ vs Col, **(C)** NaCl + CaCl_2_ vs Col and **(D)** NaCl vs NaCl + CaCl_2_ after 7 days of germination of alfalfa seeds under NaCl, CaCl_2_ and NaCl + CaCl_2_ treatments. (The x-axis is the description of gene enrichment entries, the y-axis is the number of DEGs enriched in GO terms, and Up and Down are up and down).

GO and KEGG annotations were performed on differentially expressed DEGs in alfalfa seedlings under NaCl stress and NaCl stress with exogenous addition of CaCl_2_ to obtain possible functional information of differentially expressed DEGs. GO annotation showed that the differentially expressed DEGs in alfalfa seedlings were mainly enriched in biological processes (BP) and molecular functions (MF) under NaCl stress and exogenous addition of CaCl_2_ under NaCl stress. In NaCl vs Col, 262 DEGs were enriched in biological processes such as cellular carbohydrate metabolism, response to oxidative stress, and polysaccharide metabolism, and biological functions such as hydrolase activity, peroxidase activity, oxidoreductase activity, and antioxidant activity (**Figure 5A**); In CaCl_2_ vs Col, 552 DEGs were mainly enriched in biological processes such as stress response, lipid metabolism and molecular functions such as iron ion binding, oxidoreductase activity and transferase activity (**Figure 5B**); in NaCl + CaCl_2_ vs Col, 552 DEGs were mainly enriched in biological processes such as multicellular organisms and molecular functions such as ion binding, transcriptional regulator activity, oxidoreductase activity, DNA binding transcription factor activity and enzyme binding (**Figure 5C**); In NaCl vs NaCl + CaCl_2_,323 DEGs were mainly enriched in biological processes such as multi-cellular biological processes and multi-organism processes and molecular functions such as oxidoreductase activity, hydrolase activity, transferase activity and iron ion binding (**Figure 5D**). It indicated that multicellular biological processes, oxidoreductase activity and hydrolase activity may be involved in the regulation of growth and development of alfalfa root tip cells under NaCl stress and NaCl stress with exogenous addition of CaCl_2_.

**Figure 6:**
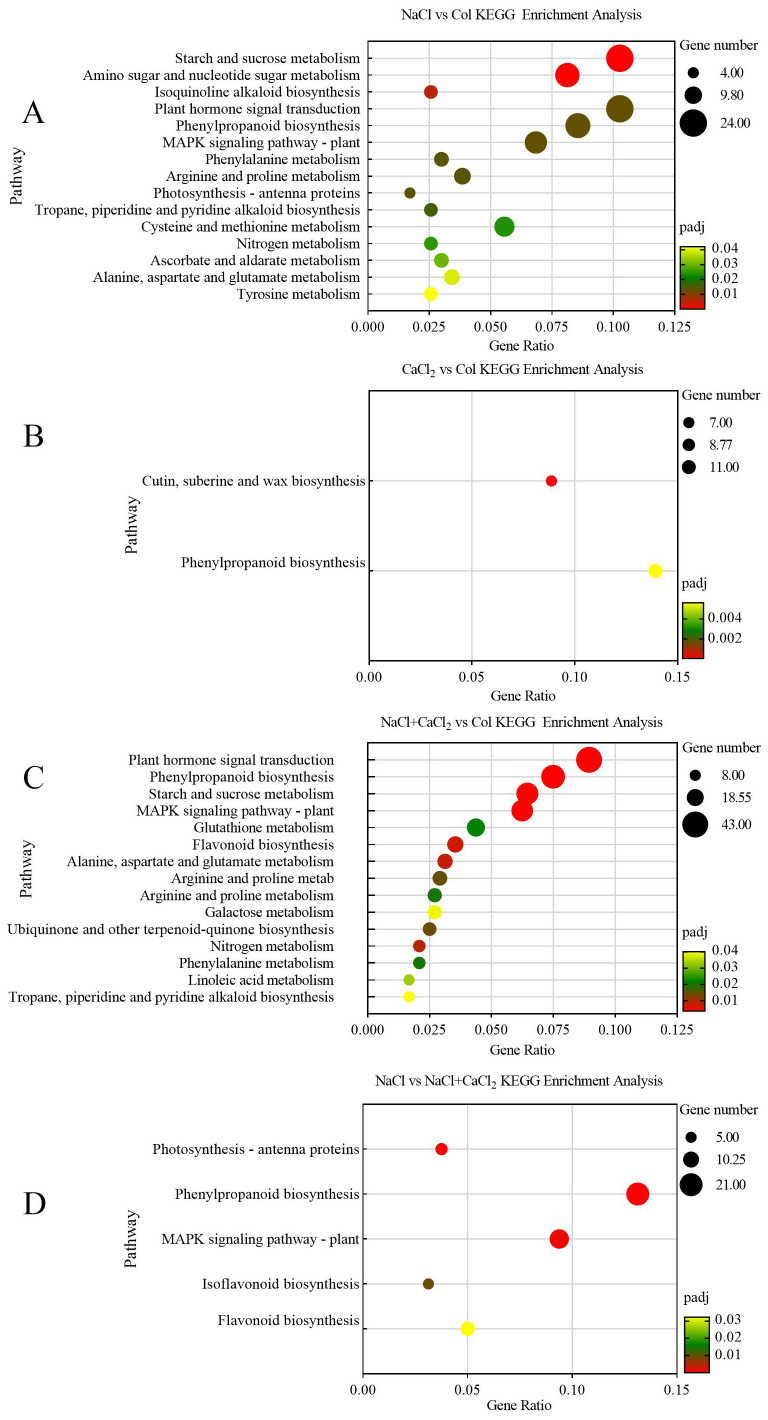
KEGG enrichment analysis of differentially expressed DEGs in **(A)** NaCl vs Col, **(B)** CaCl_2_ vs Col, **(C)** NaCl + CaCl_2_ vs Col and **(D)** NaCl vs NaCl + CaCl_2_ after 7 days of germination of alfalfa seeds under NaCl, CaCl_2_ and NaCl + CaCl_2_ treatments. The x-axis is the ratio of the number of differentially expressed genes annotated to the KEGG pathway to the total number of differentially expressed genes. The y-axis is the name of the KEGG pathway. The bubble size indicates the number of enriched DEGs, and the color indicates the significance level).

Based on the obtained KEGG annotation information of differential DEGs, the biological pathways enriched by differential DEGs were further revealed. When alfalfa seeds germinated for 7 days under NaCl, CaCl_2_, and NaCl + CaCl_2_ treatments, 175 DEGs were significantly enriched in 15 pathways such as starch and sucrose metabolism under NaCl vs Col (Padj< 0.05), of which 49 DEGs were significantly enriched in starch and sucrose metabolism, amino sugar and nucleotide sugar metabolism, and isoquinoline alkaloid biosynthesis pathways Padj< 0.01) (**Figure 6A**). As shown in **Figure 6B**, there was phenylpropanoid biosynthesis under CaCl_2_ vs Col, and 18 DEGs were significantly enriched in the biosynthetic pathways of cutin, palmitate and wax (Padj< 0.05). Under NaCl + CaCl_2_ vs Col, 281 DEGs were significantly enriched in 15 pathways such as plant hormone signal transduction, phenylpropanoid biosynthesis, starch and sucrose metabolism (Padj< 0.05), of which 140 DEGs were significantly enriched in plant hormone signal transduction, phenylpropanoid biosynthesis, starch and sucrose metabolism, MAPK signal transduction 4 pathways (Padj< 0.01) (**Figure 6C**). Under NaCl vs NaCl + CaCl_2_, 55 DEGs were significantly enriched in 5 pathways of photosynthesis-antenna protein, phenylpropanoid biosynthesis, plant MAPK signal transduction and isoflavone biosynthesis (Padj< 0.05), of which 42 DEGs were significantly enriched in 3 pathways of phenylpropanoid biosynthesis, plant MAPK signal transduction and photosynthesis-antenna protein (Padj< 0.01) (**Figure 6D**). It can be seen that when exogenous CaCl_2_ was added under salt stress, the differentially expressed genes mainly responded to the alleviation effect of CaCl_2_ on alfalfa salt stress in phenylpropanoid biosynthesis, plant MAPK signal transduction and photosynthesis-antenna protein pathway.

### Analysis of differential gene regulatory network of alfalfa after adding CaCl_2_ under salt stress

**Figure 7:**
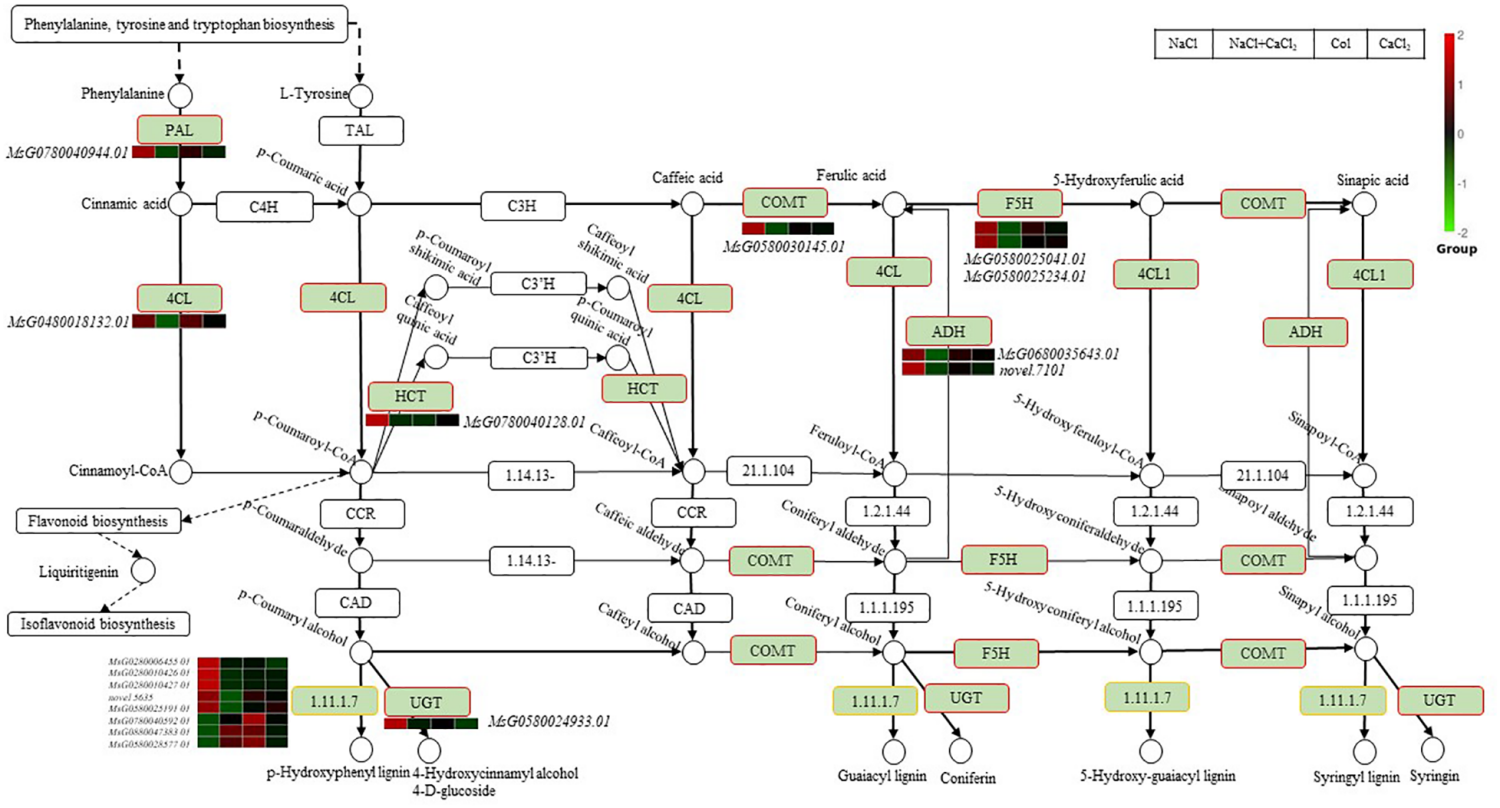
Regulation network diagram of differentially expressed DEGs in phenylpropanoid biosynthesis pathway in alfalfa seeds treated with NaCl, CaCl_2_ and NaCl + CaCl_2_ for 7 days after germination. (According to the KEGG database and related literature, the pathway network diagram was drawn. The red border was the KEGG node of the up-regulated gene, and the yellow border was the KEGG node containing the up-regulated and down-regulated genes).

In this study, we found that alfalfa uses the phenylpropanoid biosynthesis pathway to defend against salt stress. There were 21 differentially expressed genes in the phenylpropanoid biosynthesis pathway of alfalfa seedlings after adding CaCl_2_ under salt stress (**Figure 7**). The enriched differential genes were phenylalanine ammonia lyase (PAL, MsG0780040944.01), 4-coumarate-CoA ligase (4CL, MsG0480018132.01), hydroxycinnamoyl transferase (HCT, MsG0780040128.01), caffeic acid O-methyltransferase (COMT, MsG0580030145.01), ferulic acid 5-hydroxylase (F5H, MsG0580025041.01,MsG0580025234.01).Aldehydedehydrogenase(ADH,MsG0680035643.01,novel.7101),UDPglycosyltransferase(UDP,MsG0580024933.01),peroxidase(MsG0280006455.01,MsG0280010426.01,MsG0280010427.01,novel.5635,MsG0580025191.01,MsG0880047383.01,MsG0780040592.01).The unidentified protein LOC112420308 (MsG0580028577.01) and the metabolites in the phenylpropanoid biosynthesis pathway p-coumaric acid coenzyme A involved in the flavonoid and isoflavone biosynthesis pathway gene β-glucosidase (BGL, MsG0680034978.01, MsG0480021135.01),iso liquiritigenin 2 ‘ -O-methyltransferase(CHMT, MsG0180001447.01, MsG0180001403.01). Among the genes differentially expressed in the phenylpropanoid biosynthesis pathway, peroxidase (MsG0880047383.01, MsG0780040592.01) up-regulated the synthesis of lignin under NaCl + CaCl_2_ treatment.

**Figure 8:**
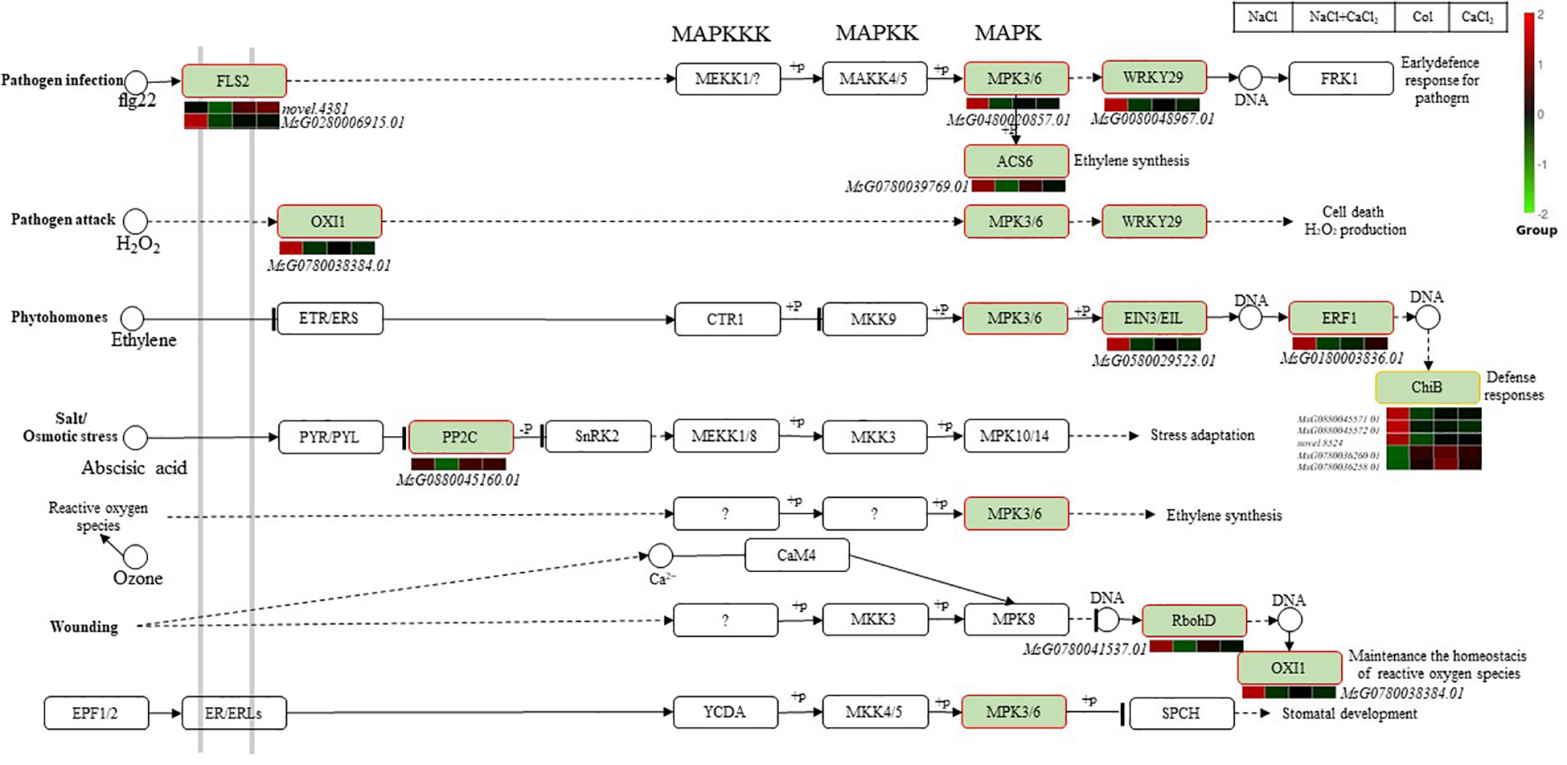
Regulation network diagram of differentially expressed DEGs in MAPK signal transduction in alfalfa seeds treated with NaCl, CaCl_2_ and NaCl + CaCl_2_ for 7 days after germination. (According to the KEGG database and related literature, the pathway network diagram was drawn. The red border was the KEGG node of the up-regulated gene, and the yellow border was the KEGG node containing the up-regulated and down-regulated genes).

In the MAPK signal transduction pathway, the MAPK cascade consists of at least one MAPK, one MAPK kinase (MAPKK) and one MAPKK kinase (MAPKKK) (**Figure 8**). In this study, it was found that 15 differentially expressed genes were significantly enriched in the MAPK signal transduction pathway of alfalfa seedlings treated with CaCl_2_ under salt stress, in the pathogen infection pathway, two genes (novel.4381, MsG0280006915.01) encoding LRR receptor-like serine/threonine protein kinase At3g47570 (FLS2), one encoding MPK3/6 gene mitogen-activated protein kinase 3 (MsG0480020857.01), and one encoding possible WRKY transcription factor 29 gene (WRKY29,MsG0080048967.01) regulate early virus defense and regulate plant stomatal development. A gene encoding 1-aminocyclopropane-1-carboxylic acid synthase (ACS6, MsG0780039769.01) regulates ethylene synthesis. In the pathogen attack pathway, serine/threonine protein kinase OXI1 (MsG0780038384.01) regulates the accumulation of H_2_O_2_ and maintains the homeostasis of reactive oxygen species. In the plant hormone ethylene transduction pathway, one ethylene response transcription factor 1B (MsG0180003836.01) encoding ERF1, one gene encoding EIN3/EIL (MsG0580029523.01) and five plant chitinases encoding ChiB(MsG0880045571.01,MsG0880045572.01,novel.8524,MsG0780036260.01,MsG0780036258.01) are involved in the transduction of reactive oxygen species to regulate ethylene synthesis to defend against stress; protein phosphatase 2C77 (MsG0880045160.01), the gene encoding PP2C in abscisic acid transduction under salt stress, was induced to participate in stress adaptation. The differential expression of two endo chitinases (MsG0780036260.01, MsG0780036258.01) in MAPK signal transduction pathway was up-regulated under NaCl+CaCl_2_ treatment, which was involved in ethylene transduction to defense stress.

**Figure 9:**
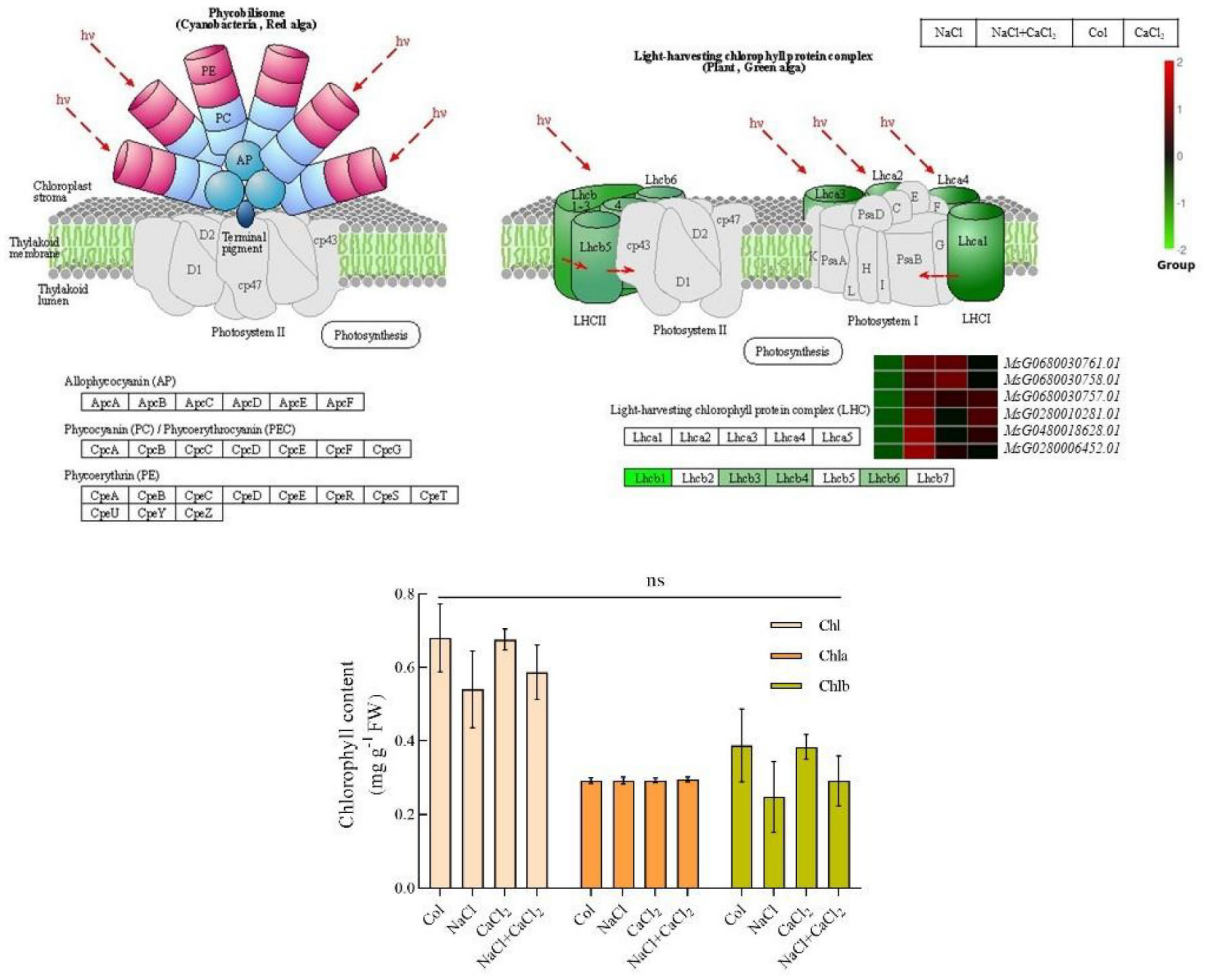
Differential DEGs expression diagram and chlorophyll content analysis diagram of photosynthesis-antenna protein pathway in alfalfa seeds treated with NaCl, CaCl_2_ and NaCl + CaCl_2_ for 7 days after germination (*Pvalue*< 0.05). (According to the KEGG database and related literature, the pathway network diagram was drawn).

The photosynthesis-antenna protein network encodes chlorophyll-binding proteins (LHC, MsG0680030761.01, MsG0680030758.01, MsG0680030757.01, MsG0280010281.01, MsG0480018628.01, MsG0280006452.01). On photosystem II, the six differentially expressed genes of LHC II1 chlorophyll ab binding protein were down-regulated under NaCl treatment, but up-regulated under NaCl + CaCl_2_ treatment. The determination of chlorophyll content under NaCl, CaCl_2_ and NaCl + CaCl_2_ treatments showed that exogenous CaCl_2_ had no significant difference in chlorophyll a, chlorophyll b and total chlorophyll content of alfalfa (**Figure 9**). It can be seen that chlorophyll binding protein plays an important role in the process of exogenous addition of CaCl_2_ to alleviate salt stress in alfalfa.

### WGCNA analysis of alfalfa after adding CaCl_2_ under salt stress

**Figure 10:**
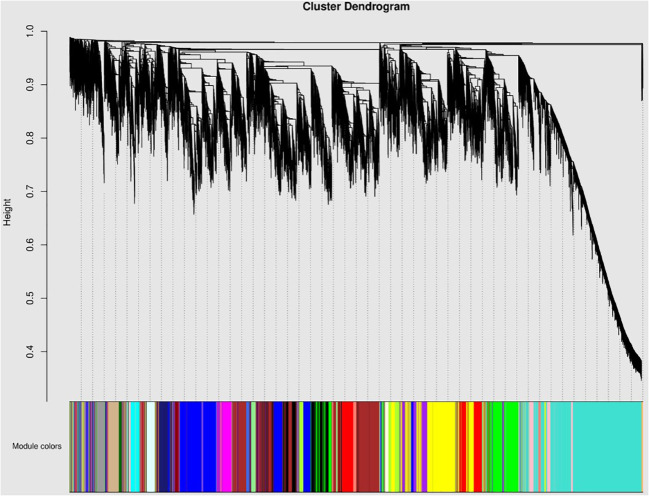
Module hierarchical clustering tree diagram. Legend: Horizontal axis: gene name or number (usually do not show the specific name, only show the cluster in order). Vertical axis: the distance of hierarchical clustering (based on TOM difference, the closer the distance, the higher the correlation of gene co-expression). Tree graph structure Branch thickness and height: Branch height reflects the co-expression difference between genes/modules. The lower the height, the higher the similarity between modules. Key branches: Identify large branches with strong co-expression signals (e.g., the main branch corresponds to the core module), or small branches independent of the main tree (which may represent specific modules).

WGCNA algorithm is a typical system biology algorithm for constructing gene co-expression network. WGCNA analysis will construct a clustering tree and divide modules according to the correlation of gene expression. Each color in the figure represents that a color corresponds to each gene on the clustering tree belonging to the same module (**Figure 10**).

**Figure 11:**
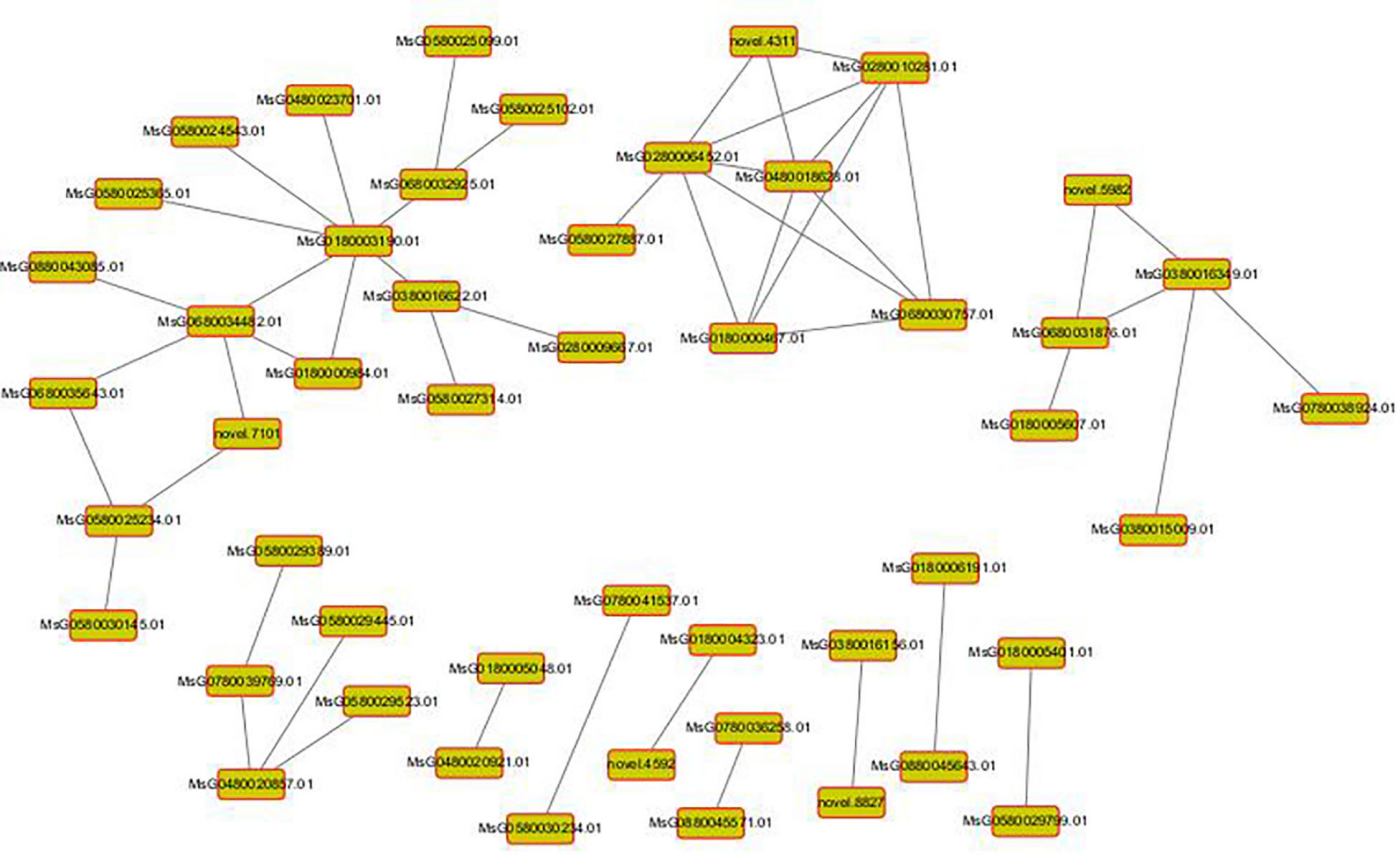
Protein interaction network diagram. Each node in the figure represents a protein, and each line represents the interaction between the connected proteins.

Then, as shown in the following figure, we apply the interaction relationship in the STRING protein interaction database to analyze the differential gene protein interaction network, and directly extract the interaction relationship of the target gene set (such as the differential gene list) from the database to construct the network. For species not contained in the database, we first apply blast to align the sequences in the target gene set to the protein sequences of the reference species contained in the STRING database, and use the protein interaction relationship of the reference species on the alignment to construct the interaction network. In the figure, the node in the interaction network: that is, an interacting protein; The degree of the node: the number of proteins interacting with this protein, the size of the degree is proportional to the core of the node, that is, the more pathways that depend on this node, the greater its core, and the greater the degree. The edge of the node: the line connecting the two nodes represents the interaction between the nodes. We performed cytoscape visualization of important gene modules or nodes and found no genes related to our research topics (**Figure 11**).

### RT-qPCR analysis

**Figure 12:**
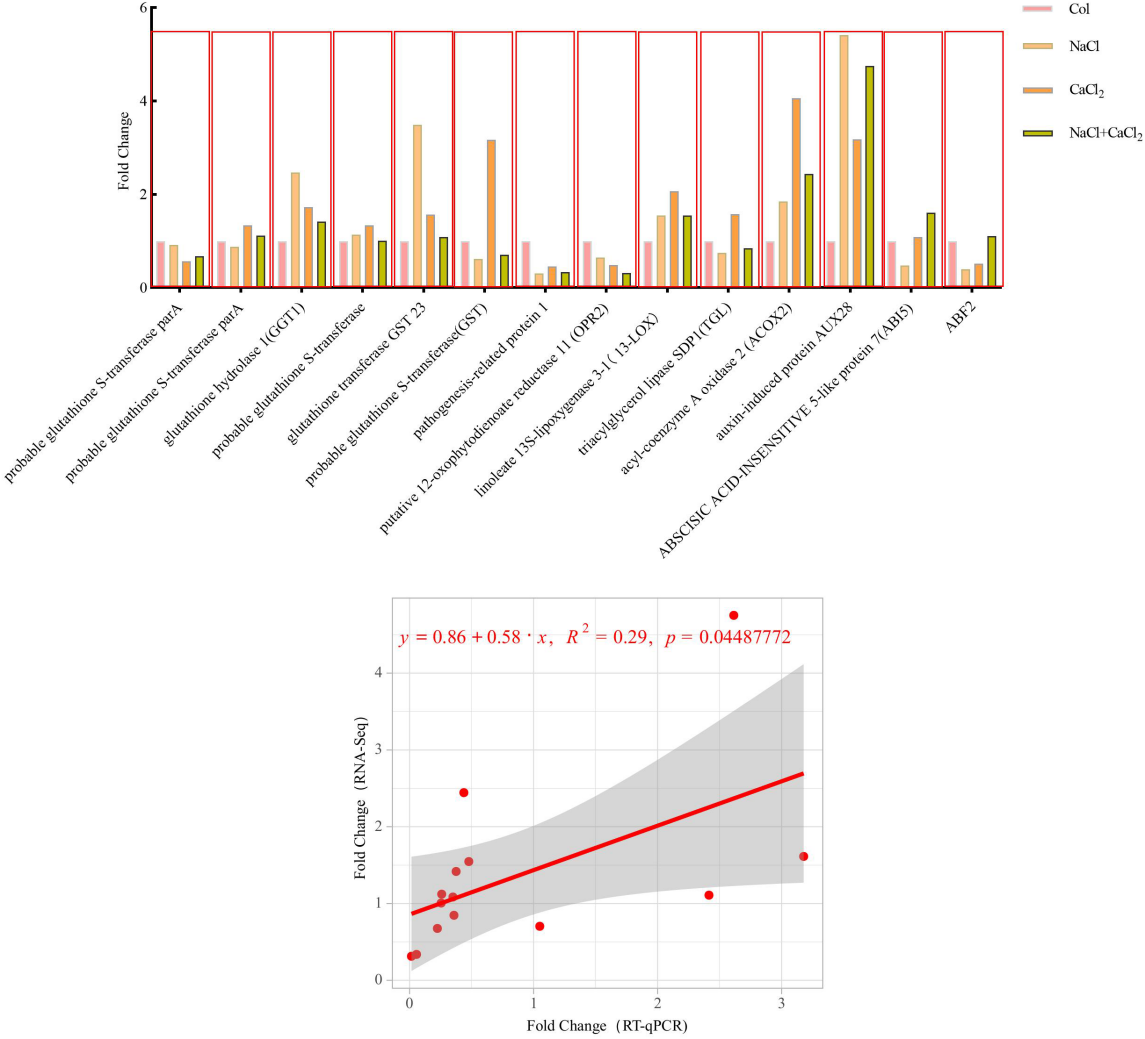
Correlation analysis between RNA-seq results and RT-qPCR results (*P*< 0.05).

RT-qPCR was used to verify the accuracy of sequencing (RNA-Seq) results, and differentially expressed genes with higher expression levels were selected from the KEGG pathway with significant changes for verification (**Figure 12**, **Supplementary Figure S2**, **Supplementary Table S7**, **S8**, RT-qPCR verification genes and primer design). The results showed that the sequencing data were highly correlated with the RT-qPCR results, and the sequencing results were reliable. It is concluded that these genes significantly expressed in the KEGG pathway are involved in the process of lignin accumulation and plant hormone transduction in alfalfa, and jointly regulate the adaptation of alfalfa under salt stress.

## Discussion and conclusions

Under saline conditions, plants mainly suffer from ion toxicity (especially Na^+^), oxidative stress caused by the accumulation of reactive oxygen species (ROS), and nutrient ion imbalance resulting from osmotic stress and ion toxicity, which in turn affect the growth and development of plant organs as well as the differentiation of plant cells (Dawood et al., 2022; Ragaey et al., 2022).

Alfalfa is a homologous tetraploid forage crop with moderate salt and alkali tolerance and high protein content (Li et al., 2011; Yang et al., 2022). In this study, under the treatment of 120 mM NaCl, the seed germination rate and seed vitality index of alfalfa significantly decreased, the elongation growth of roots was inhibited, the leaves became smaller, and the lateral growth of roots and the thickening of leaves were promoted simultaneously. The exogenous addition of CaCl_2_ had a significant mitigating effect on the NaCl stress of alfalfa, as manifested by a significant increase in the germination rate and seed vitality index, and the promotion of the elongation growth of roots and the reduction in leaf thickness. Previous studies on Chicory (*Cichorium intybus* L.), rice (*Oryza sativa*), tomato (*Solanum lycopersicum* L.), cowpea (*Vigna unguiculata*), wheat (*Triticum aestivum*), broccoli (*Brassica oleracea* var. *italica*), barley (Hordeum vulgare) also found that NaCl stress significantly inhibited the elongation growth of plants, and the seed germination rate and germination index significantly decreased (Murillo-Amador et al., 2006; Tuna et al., 2007; Arshi et al., 2010; Al-Whaibi et al., 2012; Tahjib-Ul-Arif et al., 2018; Jiang et al., 2020; Nefissi Ouertani et al., 2022; Zhang Y. et al., 2024).

When plants are subjected to salt stress, it leads to the accumulation of reactive oxygen species, oxidative stress, and cell damage. H_2_O_2_ serves as a hub for the mutual conversion of ROS. Plants reduce the damage through the antioxidant system. MDA can detect the level of lipid oxidation, and CAT and POD play important roles in the ROS scavenging system (Jiang et al., 2020; Nefissi Ouertani et al., 2022). The results of this experiment showed that the content of MDA accumulated in alfalfa under NaCl stress, and when treated with CaCl_2_, the content of MDA decreased compared with that under NaCl treatment. Studies on rice and cotton under salt stress were consistent with the results of this experiment. When under salt stress, the content of MDA increased, and under exogenous CaCl_2_ treatment, the content of MDA decreased (Guo et al., 2020; Yang et al., 2020). Under NaCl stress, the activity of CAT enzyme increased, and the activity of POD enzyme decreased. When exogenous CaCl_2_was added, the activity of CAT enzyme decreased, and the activity of POD enzyme increased. Previous studies found that when plants are subjected to salt stress, a large amount of reactive oxygen species are generated in cells. As the main component of reactive oxygen species, H_2_O_2_ will decompose in time to produce oxygen and water. The production of oxygen causes cell expansion, accelerates cell division, and may also lead to larger cells. The reason for the shortening of roots may be that the water produced by the decomposition of H_2_O_2_ participates in the metabolic process of plants, and the roots of plants will not continue to absorb the required water. At this time, the activity of peroxidase is enhanced to accelerate the decomposition of H_2_O_2_, thereby reducing the damage of oxidative stress to plant cells and defending against environmental stress (Jiang et al., 2020; Nefissi Ouertani et al., 2022).

Through the determination of the content of H_2_O_2_ in this experiment, it was found that under NaCl stress, the content of H_2_O_2_ in alfalfa decreased, the activity of POD enzyme decreased, which promoted the increase in the cross-sectional and longitudinal areas of alfalfa roots and the thickening and narrowing of leaves. When treated with CaCl_2_, compared with the treatment of NaCl, the content of H_2_O_2_ accumulated, the activity of POD enzyme increased, resulting in the reduction in the cross-sectional and longitudinal areas of alfalfa roots and the thinning and widening of leaves. We further took the root tips and leaves of alfalfa for anatomical observation. Under NaCl treatment, the cross-sectional and longitudinal areas of alfalfa roots increased, the total number of cells increased, the number of cells per unit area decreased, the area of a single cell in the cross-section increased, and the area of a single cell in the longitudinal section decreased. Anatomical observations of the leaves showed that under NaCl treatment, the alfalfa leaves became thicker and narrower, the total anatomical area decreased, and the cells became larger.

The exogenous addition of CaCl_2_ alleviated the cellular changes of alfalfa roots and leaves under NaCl stress, as manifested by the reduction in the cross-sectional and longitudinal areas of alfalfa roots, an increase in the number of cells per unit area, a decrease in the area of a single cell in the cross-section and longitudinal section, the thinning and widening of leaves, an increase in the total anatomical area, and a reduction in cell size. The high concentration of NaCl led to the imbalance of intracellular ions, and the large accumulation of Na promoted the cells to absorb more water to maintain osmotic pressure, resulting in the passive expansion of cell volume, which was manifested as the increase of single cell area in the cross section. At the same time, salt stress inhibited the expression of cell division-related genes and slowed down the rate of cell division, resulting in a slow increase in the total number of cells and a decrease in the number of cells per unit area. However, in order to maintain the absorption function of the root system, the root system will expand the contact area with the soil by increasing the cross-sectional area and longitudinal area, so as to obtain sufficient water and nutrients as much as possible. Under salt stress, in order to reduce water loss, stomatal conductance and transpiration of plants decreased, resulting in changes in the internal water state of leaves. In order to adapt to this change, the leaves reduced the water evaporation rate by thickening the cuticle and reducing the total anatomical area (narrowing). At the same time, photosynthesis is inhibited and the accumulation of photosynthetic products is reduced. However, in order to maintain the normal physiological function of cells, limited photosynthetic products are preferentially used for longitudinal elongation and thickening of cells, resulting in cell enlargement (Hao et al., 2023; Jamalirad et al., 2023; Yin et al., 2023).

Therefore, after CaCl_2_ treatment, the number of cells per unit area of alfalfa roots increased, the cell size decreased, and the root structure was more compact. This structural change is conducive to improving the absorption efficiency of water and nutrients by roots, enhancing the fixation ability of roots to soil, and thus improving the ability of plants to obtain resources under salt stress. For example, more cells mean more root hair formation, which expands the contact area between roots and soil solution, helps to absorb more water and mineral elements, and alleviates the inhibition of salt stress on plant growth. CaCl_2_ made the leaves thinner and wider, the total anatomical area increased, and the cell size decreased, which was beneficial to improve the gas exchange and light energy capture efficiency of the leaves. The thinner leaves reduced the resistance of CO_2_ diffusion, and the larger total anatomical area and more cells increased the distribution space of chloroplasts and improved the efficiency of photosynthesis. At the same time, the recovery of cell size helps to maintain the normal structure and function of organelles such as chloroplasts, further enhance the photosynthetic capacity of plants, and provide more energy and material for the growth of plants under salt stress. The improvement of the anatomical structure of roots and leaves of alfalfa by CaCl_2_ enhanced the stress resistance of plants as a whole. The synergistic optimization of root and leaf structure and function enables plants to maintain relatively normal growth and metabolism under salt stress, and improves plant survival and biomass accumulation.

Through the analysis of KEGG pathways in the transcriptome data in this study, it was found that after the treatment of adding CaCl_2_ under NaCl stress in alfalfa, the genes in the phenylpropanoid biosynthesis pathway, MAPK signal transduction pathway, and photosynthesis - antenna protein pathway are significantly differentially expressed.

The phenylpropanoid biosynthesis pathway is an important secondary metabolic pathway in plants, which can produce a large number of secondary metabolites. Among them, lignin is a secondary metabolite with a content second only to cellulose and is also a key structural material of the plant cell wall. The accumulation of lignin can enhance the physical barrier and the mechanical strength of the cell wall of plants, which is beneficial for plants to maintain the stability of cell morphology and structure under salt stress, prevent the inflow of salt ions into cells, avoid excessive accumulation of salts, and reduce the toxicity to intracellular physiological activities (Gao et al., 2023; Ma et al., 2024). Moreover, some intermediate products in the phenylpropanoid biosynthesis pathway may have antioxidant capacity, which can help plants remove excessive reactive oxygen free radicals generated by salt stress, reduce oxidative damage, and help plants resist environmental stress (Li et al., 2022; Li T. et al., 2023; Van der Cruijsen et al., 2024).

Studies have found that the adaptation of plants to salt stress is closely related to lignin synthesis. Enzymes PALs, 4CLs, and the key enzymes COMT and F5H in lignin biosynthesis are significantly up-regulated under salt stress to regulate the accumulation of lignin, which can increase the thickness of the secondary cell wall and promote the development of vascular bundles (Wu et al., 2018; Glagoleva et al., 2022; Qin et al., 2023). In the results of this experiment, phenylalanine ammonia-lyase (PAL) converted phenylalanine (or tyrosine) from the phenylalanine, tyrosine, and tryptophan biosynthesis pathways into cinnamic acid; L-tyrosine ammonia-lyase (TAL) converted tyrosine into p-coumaric acid, and cinnamic acid 4-hydroxylase (C4H) and 4-coumaroyl-CoA ligase (4CL) converted them into cinnamoyl-CoA and p-coumaryl-CoA. p-Coumaryl-CoA participates in the flavonoid biosynthesis glycyrrhizin-mediated isoflavone biosynthesis pathway; genes related to the accumulation of lignin, glucosides, and syringin as well as the mutual conversion of caffeic acid, ferulic acid, 5-hydroxyferulic acid, and coniferyl acid (4-coumaroyl-CoA ligase 4CL, hydroxycinnamoyl transferase HCT, caffeic acid O-methyltransferase COMT, ferulic acid 5-hydroxylase F5H, aldehyde dehydrogenase ADH, UDP-sugar transferase 72E1 UDP) and peroxidase POD were induced to regulate the accumulation of the metabolite lignin. It can be inferred that when alfalfa responds to NaCl stress, the treatment of CaCl_2_ may down-regulate peroxidase POD in the phenylpropanoid biosynthesis pathway, promote the synthesis and accumulation of lignin, enhance the stability and antioxidant capacity of the cell wall, and then improves its tolerance to salt stress.

In the MAPK signal transduction pathway, the MAPK cascade, as a key signal module downstream of receptors/sensors, is encoded by a small gene family, can perceive internal and external stimuli and coordinate cellular responses, and is crucial for the normal growth and development of organisms and their adaptation to the environment (Zhang and Zhang, 2022).

The group A MAPKs represented by MPK3 and MPK6 in Arabidopsis (Arabidopsis thaliana L.) are first related to plant immunity and responses to abiotic stress and play important roles in plant growth and development. The receptor-like cytoplasmic kinase BIK1, which is a component of the immune receptor FLS2, can both positively regulate the calcium influx triggered by flg22 and directly phosphorylate the NADPH oxidase rbohD at specific sites in a calcium-independent manner to enhance ROS production, highlighting the direct role of the FLS2 complex in regulating rbohD-mediated ROS production and stomatal defense (Li et al., 2014; Zhang et al., 2018). ACS in the MAPK signal transduction pathway is a rate-limiting enzyme for catalytic reactions. Subsets of MAPKs represented by SIPK/Ntf4/WIPK in tobacco (*Nicotiana tabacum L.*) and MPK3/MPK6 in Arabidopsis will be activated under stress conditions that induce ethylene production (Han et al., 2010). The plant hormone ABA accumulates rapidly in a saline environment, controls the expression of multiple stress response genes, enhances the antioxidant defense system, and regulates the accumulation of osmotic stress compounds to enhance the ability of plants to resist environmental stress. ABA-dependent MAPK6 can be activated through OXI, up-regulating ProDH as a hypersensitive response at high H_2_O_2_ levels, and the activation of OXI jointly regulates the stress on plants with the transduction of ABA and H_2_O_2_ (Lee et al., 2022). ABA signal transduction requires receptor recognition. PP2C dephosphorylates SnRK2 to inhibit its activity. After exposure to ABA, ABA, PYR/PYL/RCAR, and PP2C form a ternary complex to inhibit the activity of PP2C, reduce the inhibition on SnRK2, and promote stress response mediation (Tomaž et al., 2022; Zhang G. et al., 2024). The plant chitinase ChiB is indispensable in the processes of ABA synthesis and ethylene synthesis (Lin et al., 2021). In this study, two genes encoding the LRR-like receptor-like serine/threonine protein kinase FLS2, the mitogen-activated protein kinase 3 encoding the MPK3/6 gene, the gene encoding the possible WRKY transcription factor 29 regulated early physiological defense, the gene encoding 1-aminocyclopropane-1-carboxylic acid synthase 1 regulated ethylene synthesis, and the serine/threonine protein kinase OXI1 regulated H_2_O_2_ accumulation to maintain the homeostasis of reactive oxygen species. In the ethylene transduction pathway of plant hormones, reactive oxygen species transduction participates in ethylene synthesis. In alfalfa, the ethylene-responsive transcription factor 1B encoding ERF1 and five chitinases encoding ChiB were induced to participate in stress adaptation under salt stress, and the transcription factor encoding ERF1 regulated stomatal development through transduction. In conclusion, the MAPK cascade can perceive stimuli and coordinate cellular responses under salt stress. The relevant MAPK genes FLS2, ACS, PP2C, and chitinase ChiB in alfalfa are activated when CaCl_2_ is added, and may mainly affect ROS accumulation and stomatal defense by up-regulating ChiB, and then regulate the synthesis pathways related to ethylene and abscisic acid to enhance the salt resistance of alfalfa.

Regulating plant photosynthesis and chlorophyll-related genes and increasing chlorophyll content and protecting the structure of chloroplasts have a positive effect on plants under salt stress (Vaghela et al., 2022). Studies have found that under salt stress, plants protect the structure of chloroplasts by regulating the expression of genes related to photosynthesis and chlorophyll, thereby protecting plants from environmental stress (Shah et al., 2024). Interestingly, through the determination of the chlorophyll content of alfalfa in this experiment, it was found that exogenous CaCl_2_ had no significant effect on the chlorophyll content. The addition of CaCl_2_ under salt stress may affect the chlorophyll-binding protein LHC in the photosynthesis - antenna protein pathway of alfalfa to alleviate the NaCl stress and changes in cell size, and the specific mechanism needs to be further explored.

**Figure 13:**
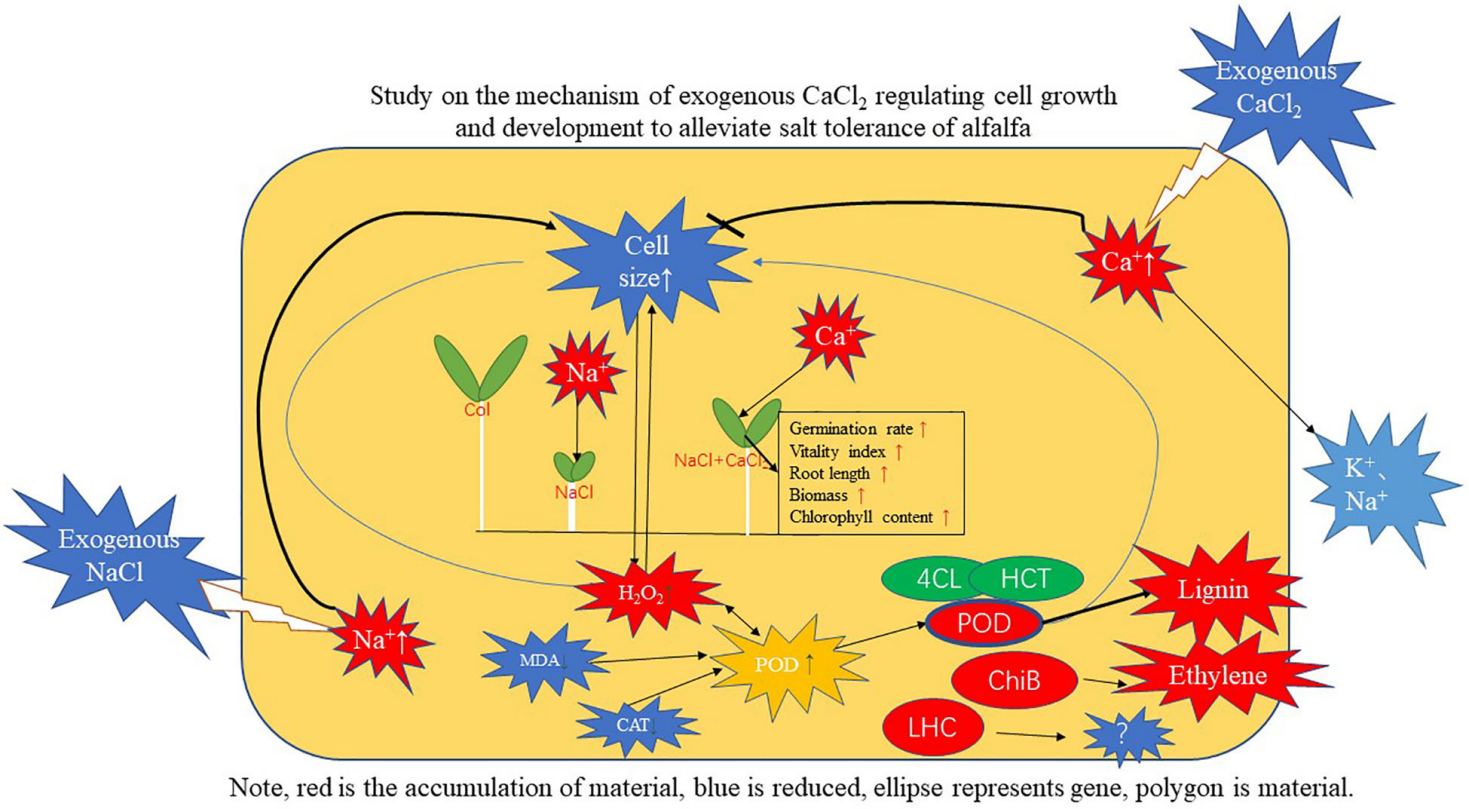
Integrating the network diagram of physiological, biochemical and genetic relationships in the results of the paper.

In summary, exogenous CaCl_2_ significantly increased the seed germination rate and vitality index of alfalfa under NaCl stress, alleviated the inhibition of NaCl stress on the elongation growth of alfalfa roots, so as to better absorb water and nutrients. Exogenous CaCl_2_ effectively alleviated the oxidative damage of membranes by alleviating the decrease in the content of H_2_O_2_, the increase in the content of MDA, the increase in the activity of CAT, and the decrease in the activity of POD in alfalfa under NaCl stress. Exogenous CaCl_2_ enhanced the resistance to NaCl stress by alleviating the enlargement of root cells and leaf cells of alfalfa under NaCl stress, reducing water evaporation and salt absorption. Transcriptome sequencing found that multicellular biological processes, oxidoreductase activity, and hydrolase activity were mainly enriched under CaCl_2_ treatment; KEGG analysis found that the addition of CaCl_2_ induced the expression of genes such as peroxidase (POD), 4-coumaroyl-CoA ligase (4CL), and hydroxycinnamoyl transferase (HCT) in the phenylpropanoid biosynthesis pathway to regulate lignin metabolism, induced the transduction of H_2_O_2_, plant hormones ethylene and abscisic acid in the MAPK signal transduction pathway of plants, and up-regulated chlorophyll-binding proteins to alleviate the NaCl stress on alfalfa (**Figure 13**).

## Data Availability

The original contributions presented in the study are publicly available. This data can be found here: https://www.ncbi.nlm.nih.gov, accession number PRJNA1279601.
