# Supplementary material for: Study on the mechanism of exogenous CaCl2 regulating cell growth and development to alleviate salt tolerance of alfalfa (Medicago sativa)
Source: Front Plant Sci. 2025 Jul 1;16:1565723. doi: 10.3389/fpls.2025.1565723 (PMC12261454; doi:10.3389/fpls.2025.1565723)
Supplement: Supplementary file 1 [file DataSheet1.pdf]

Fig.1.Effects of different concentrations of NaCl and $CaCl_2$ on alfalfa;

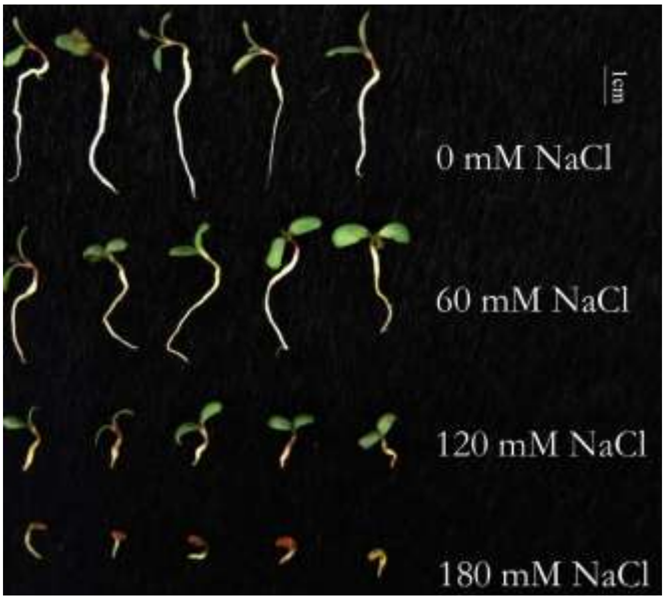

A

B

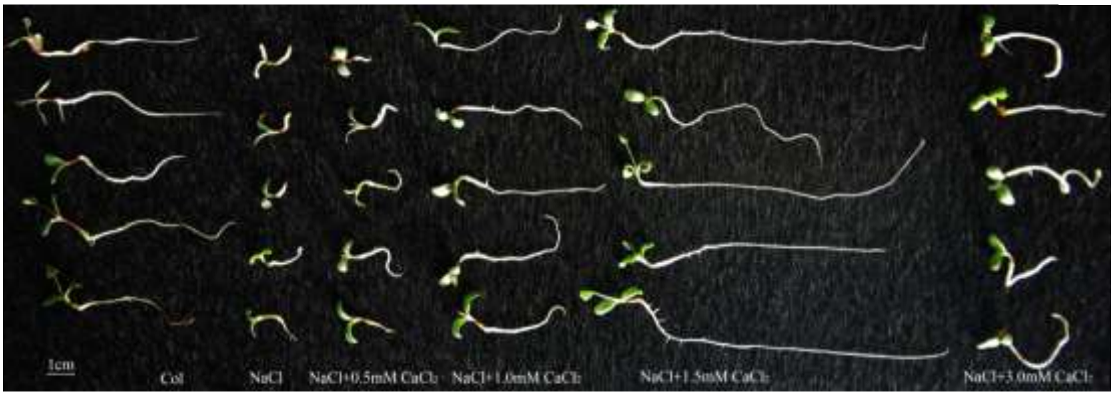

Fig.2.Agarose electrophoresis of 12 samples of alfalfa RNA

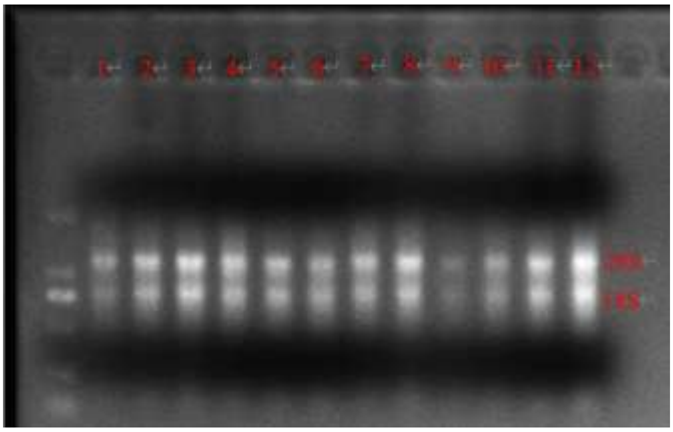

Table 1, 2, 3, 4. Real-time fluorescence quantitative PCR detection reaction system and reaction procedure

Table 1 Reverse transcription system

**Component**	**Volume**
5*FastKing-RT SuperMix	4 ul
Rnase-Free ddH_2O	14 ul
RNA	2 ul
Total	20ul

Table 2 Reverse transcription program

**Temperature**	**Time**	**Cycle number**
42℃	15 min	1
95℃	3 min	1

Table 3 RT-qPCR system

**Component**	**Volume**
2*TSINGKE Master qPCR Mix	10 ul
ddH_2O	7.4 ul
Primer-F	0.8 ul
Primer-R	0.8 ul
cDNA	1 ul
Total	20 ul

Table 4 RT-qPCR reaction procedure

**Temperature**	**Time**	**Cycle number**
95℃	1min	1
95℃	10sec	40
60℃	30sec	40

Table 5 Filtered Reads quality statistics

Sample	Raw Reads(M)	Raw Bases(G)	Clean Reads(M)	Clean Bases (G)	Q20(%)	Q30(%)
Col_1	46.04	6.91	44.64	6.7	96.38	90.54
Col_2	45.28	6.79	43.96	6.59	96.38	90.5
Col_3	46.00	6.9	45.06	6.76	96.02	89.84
NaCl_1	45.64	6.85	44.53	6.68	96.24	90.16
NaCl_2	43.75	6.56	42.71	6.41	96.44	90.67
NaCl_3	46.34	6.95	45.25	6.79	96.01	89.91
$CaCl_2$_1	46.94	7.04	45.76	6.86	96.21	90.22
$CaCl_2$_2	45.58	6.84	44.77	6.71	96.32	90.34
$CaCl_2$_3	45.24	6.79	44.41	6.66	96.33	90.35
NaCl+$CaCl_2$_1	45.46	6.82	44.41	6.66	96.12	90
NaCl+$CaCl_2$_2	45.71	6.86	43.28	6.49	96.09	89.92
NaCl+$CaCl_2$_3	44.10	6.61	42.65	6.4	96.22	90.2

Note : Raw Reads (M) : the number of reads in the original data, M represents millions ; raw Bases (G) : the number of bases of the original data ; clean Reads (M) : the number of reads after filtering the original data ; clean Bases (G) : the number of bases after filtering the original data ; q20 (%) : the percentage of bases with reads mass value greater than 20 in the total bases after filtering and the sequencing error rate was less than 1 % ; q30 (%) : The percentage of bases with a reads quality value greater than 30 in the total S bases after filtering and the sequencing error rate is less than 0.1 %.

After extracting RNA from alfalfa seedlings with different treatments, two bands of 28 S and 18 S can be seen by 1 % agarose gel electrophoresis (shown in annex figure 2). Using Agilent2100 to detect RNA, the average concentration was 423.96 ng / µl, OD260 / 280 was between 1.97 ~ 2.01, OD260 / 230 was between 1.71 ~ 2.38 (Table 1), indicating that RNA concentration and quality are high and can be used for subsequent experiments. Transcription sequencing of 12 samples yielded 79.1 G pure data and 58968 expressed genes. Each sample produced an average of 6.64 G data, and obtained an average of 43.75-46.94 (million) Raw Reads. After filtration, an average of 42.65-45.76 (million) Clean Reads were obtained, Q20 was between 96.01 % and 96.44 %, and Q30 was between 89.84 % and 90.67 %. The data show that the quality of this transcription sequencing data is relatively high and can be used for subsequent analysis.

Table 6. RNA detection results of alfalfa (' Id ' represents the detection order)

**Id**	**Sample name**	**Concentration (ng/µl)**	**A260/A280**	**A260/A230**
**1**	**Col 1**	326.48	2.00	2.12
**2**	**Col 2**	336.81	1.99	2.37
**3**	**Col 3**	464.02	2.00	2.25
**4**	**NaCl_1**	561.41	2.01	2.23

**5**	**NaCl_2**	675.34	1.97	2.29
**6**	**NaCl_3**	633.97	2.00	2.38
**7**	**$CaCl_2$_1**	175.52	1.98	1.71
**8**	**$CaCl_2$_2**	250.2	1.98	2.2
**9**	**$CaCl_2$_3**	554.27	2.01	2.25
**10**	**NaCl+$CaCl_2$_1**	425.01	2.00	2.19
**11**	**NaCl+$CaCl_2$_2**	369.79	2.00	2.29
**12**	**NaCl+$CaCl_2$_3**	314.67	1.98	2.38

Figure 3. Correlation analysis between samples and principal component analysis (PCA) results of transcriptomes of different treatments, and gene expression Venn diagram of NaCl, $CaCl_2$, NaCl + $CaCl_2$ relative to the control group.

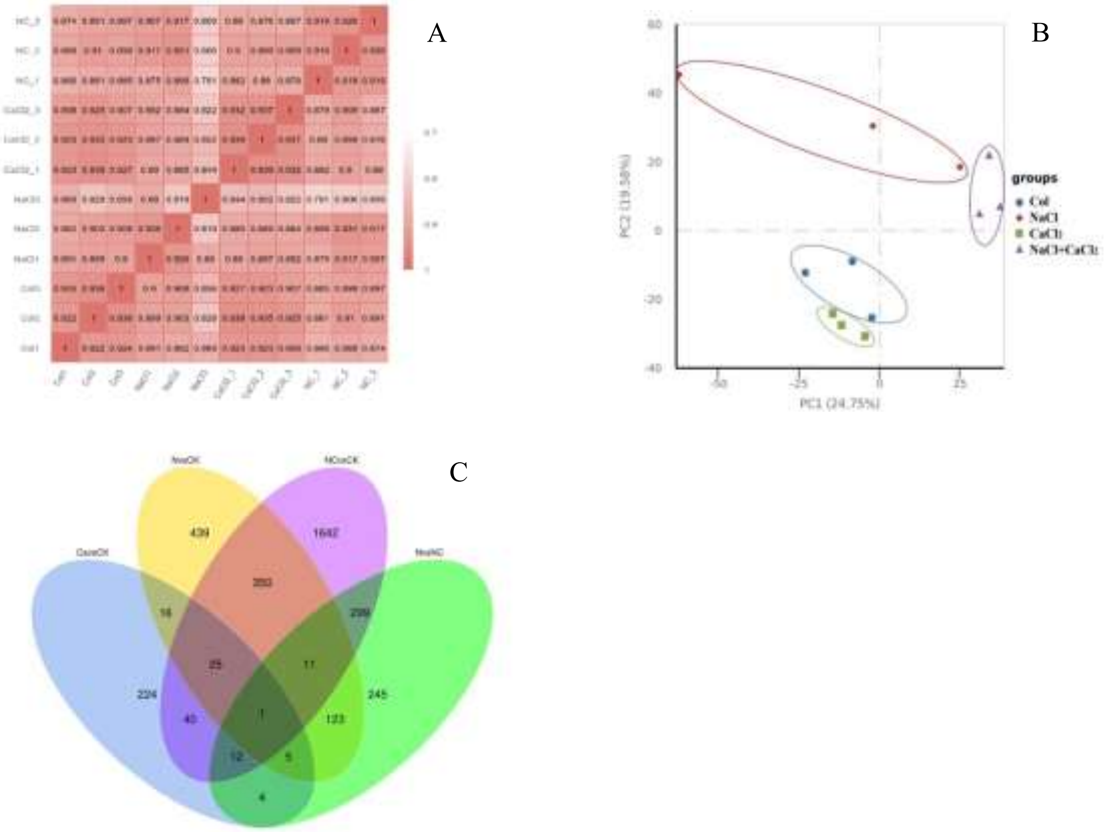

The sequencing results were highly correlated with $R^2 > 0.92$, and the correlation between the groups was between 0.828 and 0.939, indicating that the biological duplicate data were reliable. Principal component analysis (PCA) showed that the PCA values of NaCl treatment samples were significantly discrete with the other three treatments. The PCA values of NaCl + $CaCl_2$ treatment were separated in the PC2 direction and concentrated in the PC1 direction, indicating that NaCl + $CaCl_2$ treatment was significantly different at the transcriptional level. In the Venn diagram, N represents NaCl treatment, NC represents NaCl + $CaCl_2$ treatment; the number of Venn diagram overlapping genes between different treatments relative to the control group was less, indicating that they caused different gene expression changes.

Table 7. RT-qPCR validation of genes

gene_id	gene_name	tf_family	Simple name
**MsG0380015008.01**	*probable glutathione S-transferase parA*	GST_N	GST1
**MsG0380015009.01**	*probable glutathione S-transferase parA*	GST_N	GST2
**MsG0380016349.01**	*glutathione hydrolase 1(GGT1)*		GGT
**MsG0580024232.01**	*probable glutathione S-transferase*	GST_N	GST3
**MsG0480023109.01**	*glutathione transferase GST 23*	GST_N	GST4
**MsG0780038924.01**	*probable glutathione S-transferase*(GST)	GST_N	GST5
**MsG0280007934.01**	*pathogenesis-related protein 1*	SCP	SCP
**MsG0180000008.01**	*putative 12-oxophytodienoate reductase 11 (OPR2)*	Oxidored_FMN	OPR2
**MsG0880042750.01**	*linoleate 13S-lipoxygenase 3-1（13-LOX）*	Lipoxygenase	13-LOX
**MsG0780040405.01**	*triacylglycerol lipase SDP1(TGL)*		TGL
**MsG0480023339.01**	*acyl-coenzyme A oxidase 2 (ACOX2)*		ACOX2
**MsG0780040694.01**	*auxin-induced protein AUX28*		AUX28
**MsG0080048073.01**	*ABSCISIC ACID-INSENSITIVE 5-like protein 7(ABI5)*	bZIP	ABI5
**MsG0380017145.01**	*ABF2*	bZIP	ABF2

Table S8. qRT-PCR verification gene primer design

Gene Id	Primer	Gene Sequence（5’-3’）
**Reference genes**	Actin-F	CAAAAGATGGCAGATGCTGAGGAT
	Actin-R	CATGACACCAGTATGACGAGGTCG
**MsG0380015008.01**	GST1-1 F	TATGGACAGGAAAGGGTGAA
	GST1-1 R	TTGTGAGGGTGAGGAAGTGA
**MsG0380015009.01**	GST2-1 F	CTTTGGGTATGTTGATGTGG
	GST2-1 R	TTGTGAGGGTGAGGAAGTGA
**MsG0380016349.01**	GGT-3 F	GCCTTGCTGGTGGGACAATA
	GGT-3 R	AAAGCGATCAAACTCGGGAC
**MsG0580024232.01**	GST3-3 F	ATTTGTTTCTCCGACCCTCT
	GST3-3 R	TTTCCTCCAACCCTTCTAAC

**MsG0480023109.01**	GST4-1 F	TTTTGCCTCAACATCCATAT
	GST4-1 R	AAGCCAGTAAGAAATCCATC
**MsG0780038924.01**	GST5-2 F	ATGGCATCAAATAAGGAAGA
	GST5-2 R	TGTAAGGGTCAGAAGGCAAG
**MsG0280007934.01**	SCP-1F	CCCCAATGATCTCACATCTC
	SCP-1R	TACCTTAGCACATCCAACCC
**MsG0180000008.01**	OPR2-1F	GGCATTTGGACAAGGGAACA
	OPR2-1R	GCTTAATCGGCGAGGAGGTG
**MsG0880042750.01**	13-LOX-1F	TCCGAATCAATCAATGTAGG
	13-LOX-1R	CAGATGAATGAGTCGAAAGC
**MsG0780040405.01**	TGL-1F	CATCGCCTCATGTCGTTATA
	TGL-1R	GAGCCTTCTTCGGGACCTAG
**MsG0480023339.01**	ACOX2-3F	CTTCAACGCTAATCCACATC
	ACOX2-3R	AGGTTTAACTCCAGCTTCTC
**MsG0780040694.01**	AUX28-2F	ATTCTGATTATGTCCCAACC
	AUX28-2R	CAATAGCCTCCTTTCCTTTC
**MsG0080048073.01**	ABI5-1F	TAAGGAGTCGAATAAGGGAA
	ABI5-1R	TATGCTGGGATCATGTTGTG
**MsG0380017145.01**	ABF2-1F	GGTCGGTTTGGTGTTATGTG
	ABF2-1R	GAGTGGGTTTCAGTTTGGTA
